# SSPIM: a beam shaping toolbox for structured selective plane illumination microscopy

**DOI:** 10.1038/s41598-018-28389-8

**Published:** 2018-07-03

**Authors:** Mostafa Aakhte, Ehsan A. Akhlaghi, H.-Arno J. Müller

**Affiliations:** 1 Institute for Biology, Division of Developmental Genetics, University of Kassel, Heinrich-Plett Str. 40, 34132 Kassel, Germany; 2 Department of Physics, Institute for Advanced Studies in Basic Sciences (IASBS), Zanjan, 45137-66731 Iran; 3 Optics Research Center, Institute for Advanced Studies in Basic Sciences (IASBS), Zanjan, 45137-66731 Iran

## Abstract

Selective plane illumination microscopy (SPIM) represents a preferred method in dynamic tissue imaging, because it combines high spatiotemporal resolution with low phototoxicity. The OpenSPIM system was developed to provide an accessible and flexible microscope set-up for non-specialist users. Here, we report Structured SPIM (SSPIM), which offers an open-source, user-friendly and compact toolbox for beam shaping to be applied within the OpenSPIM platform. SSPIM is able to generate digital patterns for a wide range of illumination beams including static and spherical Gaussian beams, Bessel beams and Airy beams by controlling the pattern of a Spatial Light Modulator (SLM). In addition, SSPIM can produce patterns for structured illumination including incoherent and coherent array beams and tiling for all types of the supported beams. We describe the workflow of the toolbox and demonstrate its application by comparing experimental data with simulation results for a wide range of illumination beams. Finally, the capability of SSPIM is investigated by 3D imaging of *Drosophila* embryos using scanned Gaussian, Bessel and array beams. SSPIM provides an accessible toolbox to generate and optimize the desired beam patterns and helps adapting the OpenSPIM system towards a wider range of biological samples.

## Introduction

Research on the structure and function of living matter often relies upon imaging of biological samples within the µm to nm range using conventional optical microscopy. While conventional microscopes record two-dimensional images of a sample, biological processes like the behaviour and the interaction of cells in developing embryos happen within a three-dimensional space. Therefore, a major goal in microscopy is to develop techniques gaining high-quality three-dimensional images of relatively large samples within their natural environment.

**Figure 1:**
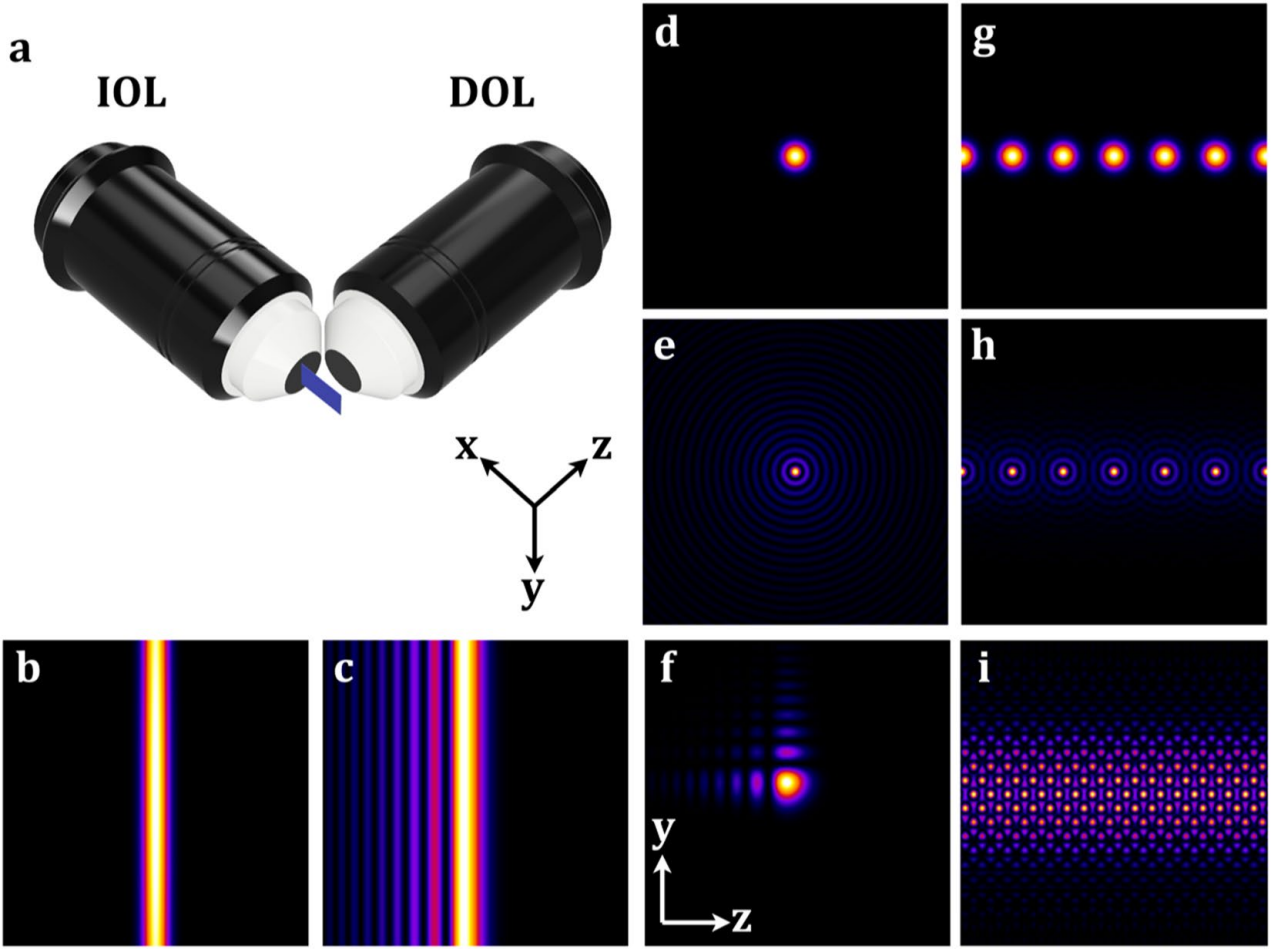
Schematic of the SPIM set up and demonstration of the illumination beams. The choice of the illumination beam depends on the size and the transparency of the biological sample. (**a**) Schematic of a conventional SPIM set up in which the illumination objective lens (IOL) generates a thin sheet within the x-y plane of the sample. The emission light is collected with the detection objective lens (DOL) in the orthogonal direction (z axis). The image was produced using the Fusion360 (Autodesk^R^). (**b**–**i**) Show simulations of the principle beam structures that can be achieved using SSPIM. (**b**,**c**) The transverse intensity of a static Gaussian beam (**b**) and of a 1D Airy beam (**c**). (**d**–**f**) Intensity profiles of a single Gaussian beam (**d**) and a Bessel beam (**e**) with radial symmetry and a 2D Airy beam (**f**) with asymmetrical intensity profile. (**g**–**i**) Beam arrays and lattice beam; (**g**) Gaussian beam array; (**h**) Bessel beam array; (**i**) Lattice beam. The simulations in (**d**–**i**) were generated using MATLAB (MathWorks^R^).

A well-known and robust method for high-resolution 3D imaging is selective plane illumination microscopy (SPIM). SPIM is based on wide-field fluorescence microscopy but applies a different arrangement of the illumination and the detection axes^1^. In SPIM, a slice of an object is illuminated by a thin sheet of light and the emitted light is collected with an objective lens in a perpendicular direction (Fig. 1a). SPIM is characterized by minimal photo bleaching and low photo toxicity, and its most attractive application represents the imaging of relatively large and living biological specimens at a high spatial and temporal resolution (Table 1). The temporal resolution is related to the imaging speed and the spatial resolution is related to the numerical aperture of the detection lens and the properties of the light sheet. Ideally, the light sheet should be narrow over a large imaging field of view (FOV) to illuminate a thin layer of the sample with an approximately equal quality along the direction of the illumination.

**Table 1:** List of biological samples which have been imaged with different type of beams.

Illumination beam/Illumination method	Biological samples
Elliptical beam (static)	*Medaka* embryo^1^, *Drosophila* embryo^1^, Zebrafish embryo^23^.
Gaussian Beam (scanning mode)	Zebrafish embryo^4^, *Drosophila* embryo^24^.
Bessel Beam (scanning mode)	mEmerald, U2OS cell, HeLa cell^5^,
1D Airy Beam (static mode)	zebrafish larva^25^, BY-2 cells, rat brain^26^.
2D Airy beam (scanning mode)	Juvenile amphioxus, renal adenocarcinoma cell, zebrafish^7^, non-cleared and cleared mouse brain^27^.
Gaussian beam array (SIM mode)	*Drosophila* embryo^12^.
Bessel beam array (SIM mode)	LLC-PK1 cell, *Dictyostelium discoideum* cell and *C*. *elegans* embryo^6^.
Lattice beam array (Dithering and SIM mode)	HeLa cell, *D. discoideum* cell, mouse embryonic stem cell, U2OS cell, mEmerald-Lifeact, *C. elegans* embryos, *Drosophila* embryo^8^.
Tiling method (Gaussian beam, Bessel beam, Lattice beam)	*C. elegans* embryo, zebrafish embryo^10,11^

The imaging FOV of a SPIM system represents the area in which the light sheet has constant intensity and thickness. Therefore, the imaging FOV is defined by the properties of the illumination beam. The first SPIM system used a cylindrical lens to produce a static light sheet (Fig. 1b)^1,2^. The penetration of the light sheet into the sample is affected by changes in the refractive index within the biological sample. The scattering of the light within the sample results in shadows, sometimes called a ‘ghost image’, that produces unwanted artefacts over the imaging FOV. In order to suppress the shadows, a pivoting method is applied in which the static light sheet is scanned in different angles within each plane using a scanning mirror^3^. Alternatively, this problem can be overcome by using different illumination beams, such as a circular Gaussian beam, which is scanned over the imaging FOV (Fig. 1d)^4^. The imaging properties of the SPIM can be further improved by more sophisticated, non-diffracting beams, such as the Airy beam or the Bessel beam.

The Bessel beam is one of the most well-known type of the non-diffracting beams^2^. Compared to the Gaussian beam, it has a narrower core and produces a larger FOV when applied to the SPIM. Like the Gaussian beam, the Bessel beam can be scanned over the imaging FOV and has been used as an illumination beam in the single- or two-photon modes of SPIM^2,5^. The application of the Bessel beam in SPIM reduces the adverse effects created by the ‘ghost image’. A disadvantage of the Bessel beam is that its core is surrounded by rings of light with a lower intensity, which results in a reduced confinement of the Bessel beam compared to the Gaussian beam (Fig. 1e). This problem can be addressed by applying a structured illumination, where instead of scanning a single Bessel beam, an array of Bessel beams is used to improve the poor confinement of the beam (Fig. 1h)^6^. Further improvements are possible by using a coherent Bessel beam array, called a lattice light sheet, which removes the rings of the Bessel beam and thereby reduces the adverse effects generated by the out-of-focus light. The lattice light sheet illumination improves axial resolution of the SPIM and greatly reduces photo bleaching and photo toxicity when compared to other methods^7^ (Fig. 1i). In addition to using Bessel beams, another approach to increase the FOV with high axial resolution has been described that makes use of an Airy beam (Fig. 1c and f). The Airy beam has a distinct intensity distribution which generates a better contrast compared to the methods that use scanning of a single Bessel beam. More importantly, the Airy beam increases the imaging FOV in SPIM even more than the Bessel beam^8^.

All types of illumination beams used for the various SPIM applications can be conveniently generated by digital beam shaping methods using a spatial light modulator (SLM). In order to inform the beam shaping, the SLM requires some kind of a digital pattern or a digital mask. In this work, we present the development of an open-source toolbox (SSPIM) for designing various digital patterns for shaping distinct beams. SSPIM supports the generation of a wide range of beams that have been routinely used in SPIM. SSPIM was designed with a graphical user interface (GUI) of MATLAB but is also able to work as a standalone. The SSPIM GUI is easy to operate for a biologist with little knowledge of spatial laser beam shaping. Our goal is to help the SPIM community to improve towards a more complete and flexible OpenSPIM system.

## Results

### Structure of the SSPIM

A major challenge that needs to be addressed in high resolution 3D imaging with SPIM is how to produce a narrow light sheet over a large imaging FOV. A narrow light sheet can be achieved by shaping the initial illumination beam. SSPIM offers a pattern generator for shaping laser beams that support all illumination modes currently used in producing the light sheets for SPIM. With the SSPIM, users are able to generate the digital pattern or mask for a static Gaussian beam, a spherical Gaussian beam, a Bessel beam, as well as the 1D and 2D Airy beams. In addition, it can produce patterns for incoherent and coherent arrays of beams. The output of the toolbox can be used by printing it e.g. via nanolithography or to control a spatial light modulator (SLM).

**Figure 2:**
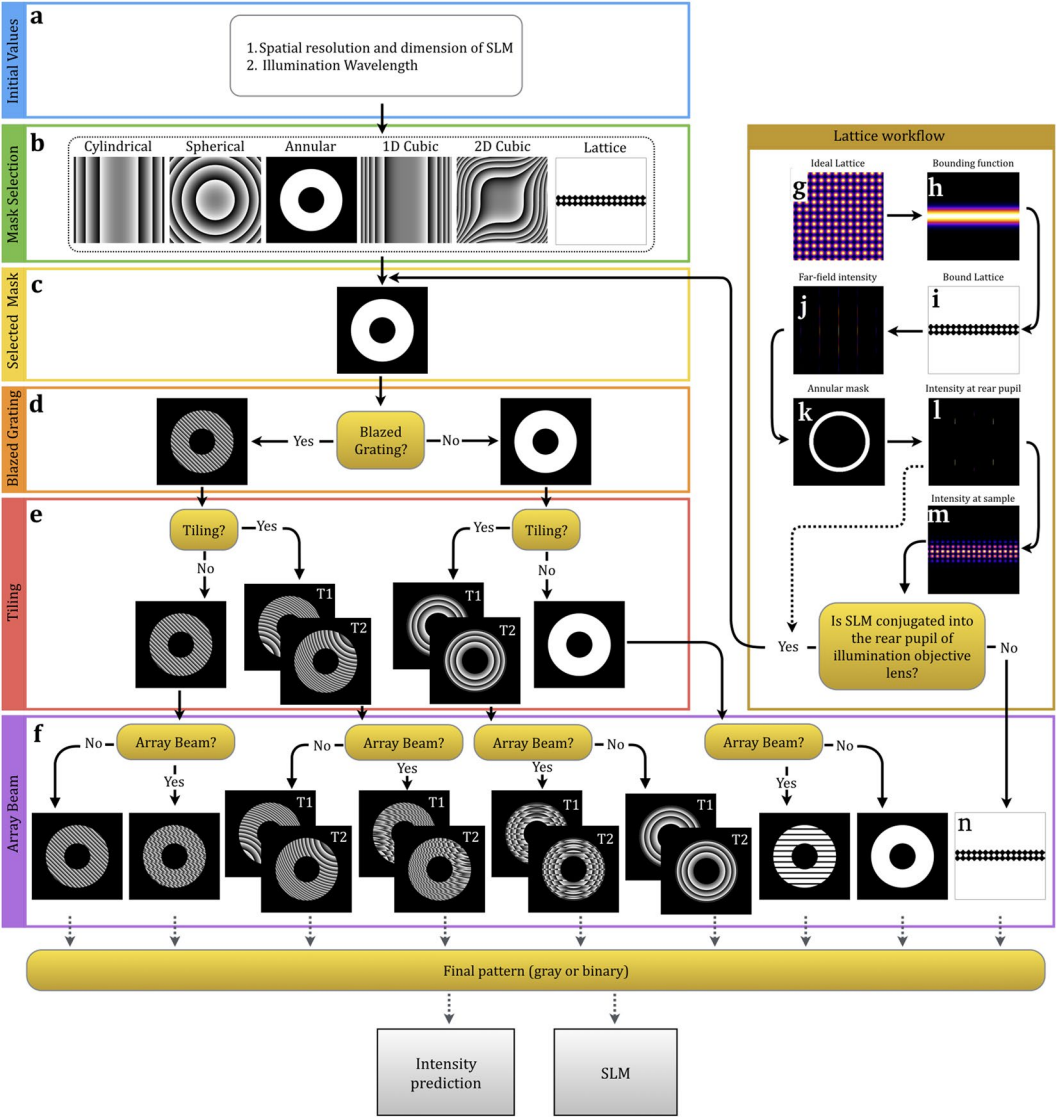
The workflow of SSPIM. A flow diagram is depicted that describes the successive steps and options in SSPIM that are available for the user when designing bespoken beam shapes. (**a**) In the first step, the user has to define the SLM parameters and the illumination wavelength. (**b**) The desired beam can be selected from six beam types using the mask selection menu. (**c**) As an example, the selected mask in this workflow is an annular mask in order to create a Bessel beam. (**d**) If the optical setup needs to separate the constant diffraction pattern of the SLM from the desired patterned hologram, a blaze grating (or binary grating for binary SLM) has to be selected. (**e**) To apply the tiling method, two or more quadratic phases can be added to the phase map; this will result in tiling the optical beam in the direction of the illumination. (**f**) In the next step, the user is able to add a binary Dammann grating to the phase map in order to generate a beam array. In step (**b**), if the user selects the “lattice”, another sub-menu (the lattice workflow) will be opened for creating an SLM pattern for a lattice beam. (**g**–**i**) In the lattice workflow, an ideal 2D lattice (square and hexagonal) (**g**), the bounding function (**h**) and the bound lattice (**i**) will be calculated. (**j**) The diffraction pattern of the bound lattice pattern will be predicted. (**k**) An annular mask selects the desired spatial frequency of the bound lattice. (**l**,**m**) The beam intensity at the rear pupil of the IOL and in front of the focal plane of the IOL will be predicted. If the SLM is conjugated into the rear pupil, the bound binary lattice (**i**) will be selected as the SLM pattern. The calculated rear pupil intensity pattern (**l**) will be set as the selected mask. Then, all steps from (**d**) to (**f**) can be processed. In general, the output of the toolbox in step (**f**) can be used as an SLM pattern for beam shaping. The user is also able to truncate the phase map with an amplitude mask. In addition, the final SLM pattern can be saved for binary or gray-scale SLM.

The work-flow of the SSPIM is composed of two major steps (Fig. 2). The first step defines the wavelength of the illumination beam and the spatial resolution of the SLM (Fig. 2a). An SLM is able to control the spatial properties of the illumination beam by applying a computer-generated mask to change the phase and the amplitude of the beam. The spatial resolution of the SLM is related to the number of active pixels per area. The second step selects the digital pattern from a range of masks to produce the desired beam structure (Fig. 2b–f). One of the following masks can be selected: (1) cylindrical, (2) spherical, (3) annular, (4) 1D cubic, (5) 2D cubic and (6) lattice (Fig. 2b). According to the selection, the mask for the desired beam will be calculated; for instance, an annular mask will yield a Bessel beam (Fig. 2c).

The next step in the workflow takes into account an unwanted, constant diffraction pattern based on the inherent structure of an SLM. Since the centre of this constant diffraction pattern is exactly on the optical axis, it disturbs the outgoing beam structure and therefore needs to be eliminated. One effective method to separate the desired beam from the unwanted diffraction pattern is to introduce an asymmetry into the selected mask. This antisymmetric mask generates an off-axis optical beam path, which diverts the desired beam away from the unwanted constant diffraction pattern of the SLM. For this reason, we implemented a blaze grating method in SSPIM^9^. The blaze grating is a linear phase map with a specific tilt angle that can change the centre of the desired beam; by increasing the tilt angle, the desired beam will be separated from the unwanted diffraction effect (Fig. 2d).

In the next step the workflow allows the user to add a tiling option to the beam structure. In SPIM, there is a trade-off between the axial resolution and the imaging FOV: a thin light sheet provides a high axial resolution and reduces the out-of-focus light, but it generates a small FOV. In contrast, a thick light sheet provides a large FOV, but yields a lower axial resolution. To employ a uniformly thin light sheet that is still able to span the entire diameter of the specimen, a thin light sheet can be successively moved in the direction of the optical axis of the illumination objective lens (IOL)^10,11^. This method, called beam tiling, allows the light sheet to be swept along the direction of the illuminating light sheet in a step-wise fashion. For instance, if the effective FOV of the light sheet is 25 μm and the desired FOV is 100 μm, the thin light sheet should be moved four times with 25 μm step size. One way to achieve tiling is by successively changing the focus position of the light sheet using multiple spherical lenses with different focal lengths; however, changing multiple lenses mechanically is too slow for high speed imaging. We addressed this problem by applying a digital spherical lens that is controlled by the high speed SLM to produce different focal lengths (Fig. 2e). This tiling method can be applied in all light sheet illumination modes and provides a robust approach to achieve imaging at the sub-cellular resolution.

In the final step, SSPIM provides the option to include structured illumination methods to the beam pattern. Structured illumination microscopy (SIM) approaches are employed to improve the contrast and the spatial resolution of SPIM. One of the principal methods to generate the beam arrays for SIM light sheets uses an SLM^6^. In order to create a beam array using a spatial modulation method we need an optical element that makes ‘n’ copies of the illumination beam. In SSPIM, this is achieved by using Dammann and optimal gratings. These are diffractive optical elements, which split the incoming beam into a fan of ‘n’ outgoing beams with approximately similar intensities^12–16^. The number of the outgoing beams is adjusted with the transition number of the Dammann grating, and the angular spacing of the outgoing beams is determined by the spatial frequency of the Dammann grating (Fig. 2f). The mask that was created in the previous steps for a single beam is merged with the Dammann or the optimal grating to generate an incoherent beam array.

The lattice beam provides a powerful structured illumination beam in SPIM for high-resolution imaging with minimal photo bleaching over a large FOV. The high structural confinement of the lattice beam is based on the reduction of out-of-focus light, because the energy of the laser beam is spread out homogenously between multiple beams. This structured beam can be used in two modes of the light sheet illumination: a scanning mode and a SIM mode^8^. With SSPIM the user designs a diffractive pattern for a lattice beam in three steps: First, the 2D structure of the lattice is defined by setting the size of the illumination beams and the distances between the individual beams of the array; SSPIM supports the formation of square or hexagonal 2D lattice patterns (Fig. 2g). In the second step the 2D lattice is transformed into a 1D lattice using a bounding function; the bounding function is a Gaussian function that is elongated in one direction and is bound in orthogonal direction (Fig. 2h). In the third step, the intensity pattern of the 1D lattice (Fig. 2j) is adjusted with an annular mask; the annular mask filters the spatial frequencies of the 1D lattice and thereby modifies the properties of its intensity (Fig. 2k–l). The intensity at this step shows the intensity at the rear pupil of the illumination objective lens (Fig. 2l). Finally, the intensity of the lattice beam that was generated through the designed pattern is simulated at the sample plane and depicted in the SSPIM user interface (Fig. 2m).

### Spatial Laser beam shaping using SSPIM

The control of the spatial shape of a laser beam requires to modulate the phase or the amplitude of the light. In this study, we developed SSPIM as a tool to create optical masks using an SLM, which supports flexible and fast light modulation. While SSPIM is designed for gray scale and for binary SLM, in our set up we used a binary SLM, which is conjugated into the back focal plane of the illumination lens (Suppl. Mat. Fig S1a). To evaluate the ability of SSPIM to generate different patterns for beam shaping we measured the beam intensity in cross-section and over the FOV. These measurements were conducted by recording the beam propagation through a dye solution. We will describe the results of these measurements and the adjustments required to optimize the different beam shapes in the following.

#### Static Gaussian beam and 1D Airy beam

**Figure 3:**
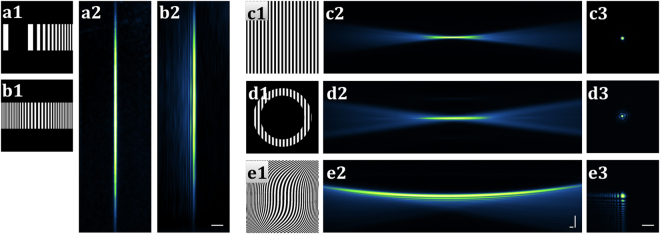
SSPIM output for single beams. (**a**,**b**) The binary pattern and recorded cross-section intensities of the static Gaussian beam (**a1**,**a2**) and 1D Airy beam (**b1**,**b2**) are depicted, respectively. (**c1**,**d1**,**e1**) The calculated SLM patterns to create the circular Gaussian beam (**c**), the Bessel beam (**d**) and the 2D Airy beam (**e**). (**c2**,**d2**,**e2**) Measurements of the beam propagation intensity through a dye solution. (**c3**,**d3**,**e3**) Recorded cross–sections of the beams. Scale bars: 20 μm.

The static Gaussian beam and the 1D Airy beam were produced by quadratic and cubic phase modulations, respectively (Fig. 3a1 and b1). These phase modulations have to be adjusted for the fine-tuning of the thickness and the imaging FOV of the desired beams. The static Gaussian beam is adjusted with the rectangular mask. The adjustment of the 1D Airy beam in y direction (Fig. 1c) is controlled by the cubic phase coefficient and the size of the rectangular mask (see Methods).

#### Gaussian beam

The most commonly used illumination techniques in conventional SPIM systems apply a scanning Gaussian beam with circular symmetry^4^. The modulation of the Gaussian beam with an SLM requires an optical element to separate the default SLM diffraction pattern from the Gaussian beam (see above). For this reason, blaze grating is implemented into the SSPIM to select the desired Gaussian beam (Fig. 3c1–c3). The imaging FOV of the separated beam can be adjusted with a circular mask (Suppl. Mat. Fig. S2). The circular mask is a digital aperture and by decreasing its radius (Supp. Mat. Fig. S2. a1–d1) the numerical aperture of the Gaussian beam is decreased which results in a large imaging FOV and a thick beam (Suppl. Mat. Fig. S2 a2–d2).

#### Bessel beam

To improve the resolution of the imaging of a large specimen with SPIM, SSPIM offers the ability to employ Bessel beams using annular masks (Fig. 3d1–d3). The properties of the Bessel beam can be adjusted by changing the inner radius and the outer radius of the annular mask (Suppl. Mat. Fig. S3). The core size of the Bessel beam and the number of the surrounding rings determine the axial resolution, the contrast and the FOV of the imaging. A smaller core and a higher number of the surrounding rings are achieved by increasing the inner and outer radii of the mask proportionally (Suppl. Mat. Fig. S3a1–S3c4, S3g). Alternatively, the Bessel beam can be tuned to the same axial resolution but distinct FOVs with a constant number of rings (Suppl. Mat. Fig. S3d1–S3f4, S3h). These capabilities are critical to match the parameters of the Bessel beam with the dimensions of distinct specimens. For instance, a Bessel beam with a higher number of rings penetrates the sample better than the equivalent Bessel beam with a lower number of rings. Conversely, a Bessel beam with a lower number of surrounding rings is useful for high contrast imaging of a transparent sample.

#### Airy beam

SSPIM provides a pattern generator to optimize the application of an Airy beam to conserve the intensity of the beam along its specific propagation length. To this end, SSPIM applies a two-dimensional cubic phase for the generation of a 2D Airy beam (Fig. 3e1–e3). The properties of the Airy beam are dependent on the coefficient of the cubic phase map. SSPIM supports the application of different cubic coefficients to facilitate the generation of different Airy beams by modifying the size of the main lobe of the beam (Suppl. Mat. Fig. S4). This way, the user is able to optimize the properties of the Airy beams for a specific specimen with regard to the axial resolution and the FOV (Suppl. Mat. Movie 1).

#### Incoherent beam array

**Figure 4:**
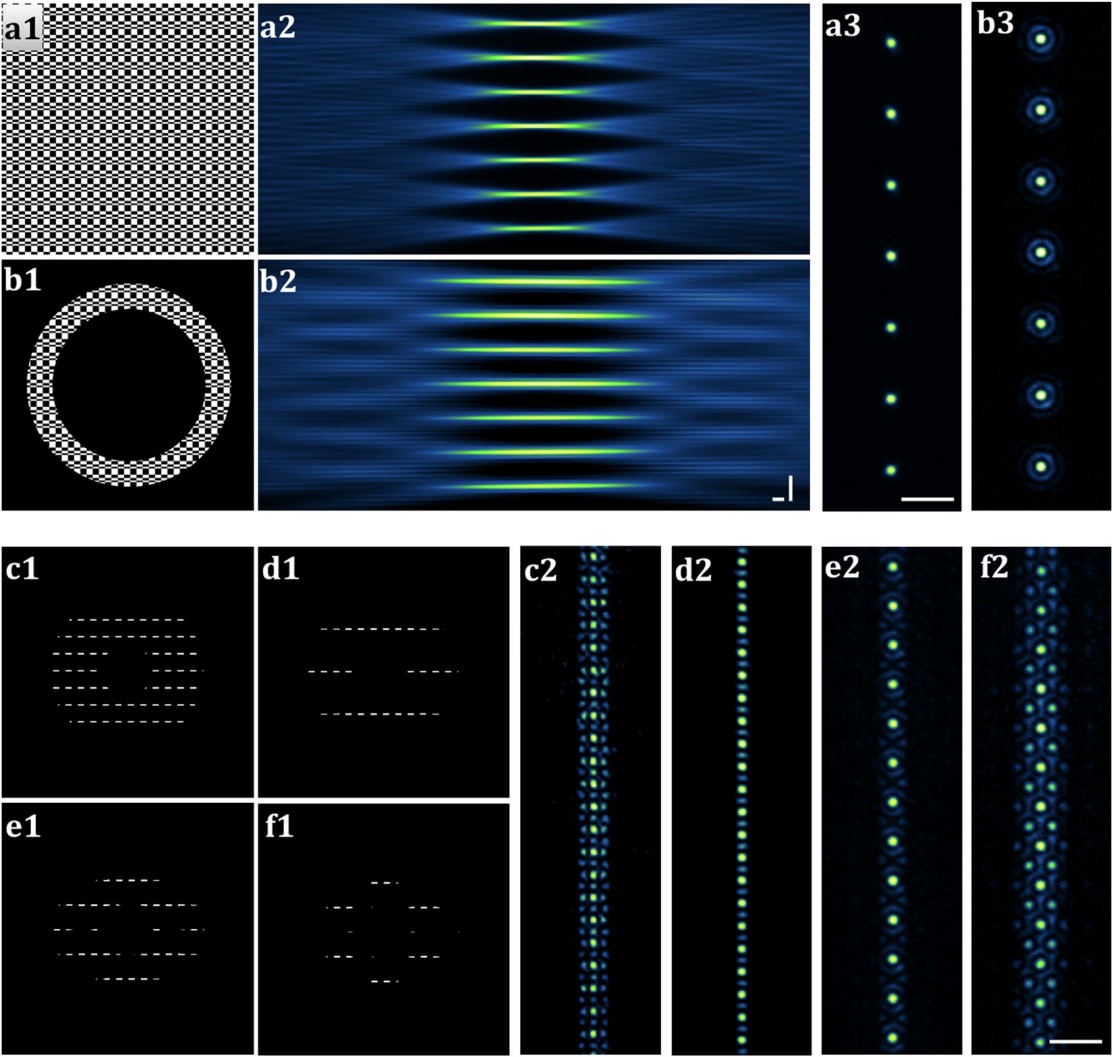
SSPIM output for array and lattice beams. (**a1**,**b1**) The binary patterns are applied to tune the SLM for the generation of the 7-array Gaussian beam (**a1**) and the 7-array Bessel beam (**b1**). (**a2**,**b2**) The recorded propagation intensity of the Gaussian beam array (**a2**) and the Bessel beam array (**b2**) through dye solution are depicted. (**a3**,**b3**) Measurements of the intensities of the beam arrays in a cross-section. (**c1**,**d1**) SLM patterns to generate the square lattice beams with different bounding functions. (**c2**,**d2**) Measurements of the intensities of the square lattice beams in a cross-section. (**e1**,**f1**) SLM pattern to generate the hexagonal lattice beams with different bounding functions. (**e2**,**f2**) Measurements of the intensities of the hexagonal lattice beams in cross-section. Scale bars: 20 μm.

SSPIM has the capability to generate multiple incoherent copies of the incident beams by a binary Dammann grating also known as a ‘fan-out element’. The user can choose between distinct Dammann gratings that support the formation of arrays composed of 2 and up to 21 individual beams. For example, a 7-Gaussian beam array is produced by a Dammann grating that has seven transitions (Fig. 4a1–a3). A Bessel beam array can also be easily achieved by merging an annular mask with a Dammann grating (Fig. 4b1–b3). The capability of the SSPIM to control the formation of beam arrays was examined by comparison of the simulated intensities of the Gaussian and Bessel beam arrays to the experimental results in which the beam propagation was recorded in dye solution (Suppl. Mat. Fig. S5 and S6). The experimental results demonstrate the robust capability of the SSPIM to shape the properties of the incident beam into the desired beam arrays. Because the outgoing beams of an incoherent beam array do not have a specific phase relationship, care must be taken to avoid any interference pattern between the beams. Therefore, the angular spacing between the beams should be sufficiently large to avoid an extensive overlap between the individual beams (Suppl. Mat. Movie 2 and 3). The angular spacing between the outgoing beams is readily adjusted by changing the spatial frequency of the Dammann grating in the SSPIM user interface.

#### Coherent beam array (lattice beam)

As mentioned above, SSPIM also supports an SLM pattern for the generation of coherent beam arrays. The lattice sub-toolbox has two options that allow the user to choose from square patterns and hexagonal patterns (Suppl. Mat. Figs. S7, S8). To evaluate the ability of this feature in SSPIM, we generated four different SLM patterns and analysed the cross-section intensity of the beams (Fig. 4c1–f2). SSPIM can produce different types of lattice SLM patterns, which can readily be employed in the conventional arrangement of an OpenSPIM system (Suppl. Mat. Fig. S1a). The user can adjust the properties of the lattice beams such as the angular spacing of the intensity spots, the size of the spots, and the beam thickness with SSPIM (Suppl. Mat. Fig. S7 and Fig. S8; Movie 3, Movie 4 and Movie 5). The results of these beam modulations are displayed at the user interface of SSPIM allowing the user to control the desired beam produced by the selected SLM pattern design. SSPIM also supports the formation of lattice beams irrespective of the conjugation of the SLM to the rear pupil of the IOL (Suppl. Mat. Fig. S1b).

### SSPIM application for tiling in deep tissue imaging

**Figure 5:**
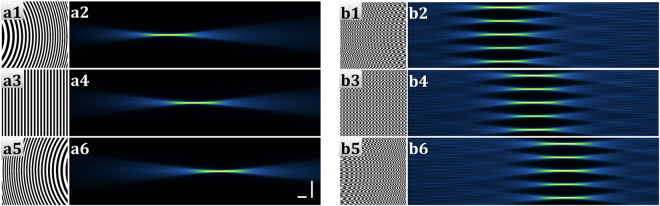
SSPIM output for the tiling method. The images provide examples for the SLM masks (**a1**,**a3**,**a5**,**b1**,**b3**,**b5**) and the measurements of the intensities of the beam propagation through a dye solution (**a2**,**a4**,**a6**, **b2**,**b4**,**b6**). (**a1**,**a3**,**a5**) SLM patterns for the application of tiling of a single beam in three steps. (**a2**,**a4**,**a6**) Measurements of the propagations of the tiled beams. (**b1**,**b3**,**b5**) SLM patterns to tiling an array composed of 5-beams in three steps. (**b2**,**b4**,**b6**) Recorded intensity propagations of the tiled beam array. Scale bars: 40 μm.

A thin optical beam with a large FOV is essential for high resolution SPIM imaging of dynamic cell and tissue movements. The tiling method described above is a relatively simple approach to address this challenge. In the tiling method, the excitation beam is swept along the direction of the light sheet propagation and thereby creates a virtual light sheet with a large imaging FOV. With SSPIM the user can apply tiling for all types of beams that we have introduced within the OpenSPIM environment. We created three-tiled holograms for the single Gaussian beam and the Gaussian beam array (Fig. 5a and b). The given imaging FOV in a sample is readily covered by tiling of an optical beam with a FOV that is smaller than the imaging FOV of the sample. The precision of the axial resolution and the enlargement of the FOV is controlled by the numerical aperture of the illumination beam and the number of tiles (Fig. 5(a1–a6)). The combination of Dammann grating with tiled patterns can produce tiled array beams which can be applied in the light sheet SIM mode (Fig. 5(b1–b6)). To examine the capability of SSPIM to support the tiling method, we created tiled holograms and measured the intensities of the different types of tiled beams (Suppl. Mat. Fig. S9). Our results demonstrate that the tiling method was applied successfully for all beam types including the Gaussian beam array, the Bessel beam array and the lattice beam. Thus, SSPIM allows users to add SIM technology over a large imaging FOV to their imaging repertoire in order to optimize the spatial resolution of the OpenSPIM system.

### SSPIM application in three-dimensional imaging with SPIM

**Figure 6:**
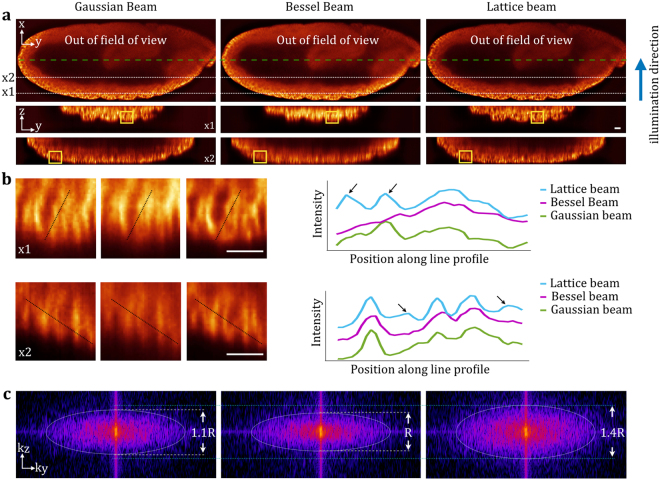
Improvement of spatial resolution in SPIM imaging with engineered illumination beams using SSPIM. *Drosophila* embryos were fixed and immunolabeled with antibodies against the membrane protein Dlg. (a, upper panels) Images of a *Drosophila* embryo recorded with SPIM using a scanned Gaussian, a Bessel beam and a scanned square lattice beam, as indicated. (a, lower panels). The axial view of the recorded data are depicted at different depths (x1 and x2). (**b**) The increased resolution using the lattice beam is demonstrated by enlarged views and the line intensity profiles of the axial views in (**a**). The graphs (right hand panels) show differences in the resolution using the three different beam types. Black arrows mark different areas in which the resolution using the lattice beam (blue line) was significantly better compared to imaging performed in the same area with either the Gaussian beam (green line) or the Bessel beam (purple line). (**c**) The spatial resolution was improved by using an engineered lattice beam. Fourier analysis of the axial view of recorded images show that the radius of the cut-off frequency is increased by using the lattice beam by approximately 40% compared to the Gaussian beam and 30% compared to the Bessel beam. Scale bars: 15 μm.

After validating the various outputs of SSPIM informed SLM generated patterns for beam shaping, we examined the capability of SSPIM to apply the desired beam patterns when imaging a biological sample. We imaged fixed *Drosophila* embryos that were immunolabeled with antibodies against the lateral plasma membrane protein Discs large (Dlg)^17^. The imaging was performed using the Gaussian, the Bessel and the square lattice light sheets, which were engineered with SSPIM in a way that the same imaging FOVs were recorded (Fig. 6). The images were compared with respect to their resolution in the axial direction. The emission intensity profile that is recorded with the camera represents the spatial information for the actual image (Fig. 6a,b). The comparison of the data confirmed that the imaging using the lattice beam light sheet can resolve more details in axial direction compared to the Gaussian or the Bessel beams (Fig. 6b). In addition, the Fourier analysis of the microscopy image data confirmed the increase in axial resolution. The recorded data using the lattice sheet illustrated a large frequency distribution, which translated into an increase in the axial resolution by approximately 40% and 30% compared to the Bessel beam and the Gaussian beam light sheets, respectively (Fig. 6c). These data demonstrate that a significant improvement in high resolution imaging was achieved by optimizing the properties of the light sheet using SSPIM.

## Conclusion

In this work, we introduced the SSPIM toolbox which was built for beam shaping within the OpenSPIM environment^18^. SSPIM is an open-source toolbox for the non-specialist user and supports all of the optical beam structures for a wide range of SPIM applications. With SSPIM, a user is able to design a digital pattern for a specific resolution, a particular imaging FOV and a specific light confinement. The desired patterns can be easily generated for binary and gray value SLMs. SSPIM predicts the beam intensity at the sample plane and presents the designed pattern as a simulation on the user interface. To examine the capability of the SSPIM in the OpenSPIM setup we have created three different SLM patterns for generating Gaussian, Bessel and lattice light sheets and tested these beams successfully for imaging a biological sample. The results show that the resolution was improved by engineering the optical beams with SSPIM. SSPIM can also work in the lattice light sheet mode within an OpenSPIM system without the need to change the structure of the microscopy setup. SSPIM will allow the user to examine different beam profiles on distinct biological samples to optimise imaging of a specific specimen using the OpenSPIM platform^18^.

## Methods

### The OpenSPIM setup

Based on the OpenSPIM system^18^, a slightly modified single illumination-detection light sheet-SPIM was built and used throughout this research (Suppl. Mat. Fig. S1a). A CW laser source (Coherent OBIS 488 nm) was used for linear excitation. The laser beam was expanded to 8.5 mm diameter and sent to a fast binary spatial light modulator (SXGA-3DM Forth Dimension Displays) for beam shaping. The binary SLM assembly consisted of a half-wave plate and a 1280 × 1024 binary SLM, which was used to modulate the wave front and the amplitude of the excitation light to generate different light sheets (Gaussian, Airy, Bessel, incoherent array or coherent lattice beams). An iris was used to block the undesired diffraction orders of the excitation light generated by the SLM. After passing the SLM, the laser beam was sent to the beam scanning mirror to create the light sheet (10 mm Galvanometer (Galvo) mirrors, Thorlabs, GVS212/M). The Galvo mirror and SLM were conjugated to the rear pupil of the illumination objective lens (Olympus UMPLFLN 10XW 0.3 NA). In order to collect imaging data from three dimensions, the sample was moved through the light sheet with a piezo (Q-522.040, PI). The emitted fluorescence was collected with a detection objective lens (Olympus UMPLFLN 20XW 0.5 NA) that was placed orthogonal to the illumination objective lens. The emitted light was detected using a sCMOS detection camera (Hamamatsu, ORCA-Flash 4.0 V2) with the help of a tube lens (U-TLU-1-2 tube) and a GFP emission filter (Thorlabs, MF525-39, BW 39 nm). To control this OpenSPIM setup, a software was built using Labview (National instruments). The imaging data were deconvolved and reconstructed with the Lucy-Richardson method using MATLAB and Fiji.

### Collection and preparation of *Drosophila* embryos

*Drosophila* flies were cultivated after standard methods and embryos were collected on yeasted apple juice plates and fixed and stained as described elsewhere^19^. The antibodies used were anti Dlg (monoclonal 4F3, Developmental Studies Hybridoma bank) and as secondary antibodies goat-anti-mouse Alexa488 (Invitrogen, Thermo Fisher Scientific, Darmstadt, Germany). The samples were embedded into a 1.5% agarose gel that was attached to a glass capillary^18^.

### Cylindrical lens phase map (Static Gaussian beam)

A static light sheet can be produced with a cylindrical lens and the transmittance function of a cylindrical thin lens can be defined as:

1 $$T(x)=\exp (i{k}_{0}\frac{{x}^{2}}{2f}).$$

Where the ${k}_{0}$, $x,$ and $f$ show the wave-number, transverse coordinate, and focal length, respectively^20^. If we assume the incident beam has plane wave, the intensity distribution in the focal plane of the cylindrical lens looks like a line. Hence, propagation of the line beam can generate a static light sheet in the Rayleigh range.

### Spherical Lens phase map (circular Gaussian beam)

In order to achieve a focused beam with Gaussian intensity distribution, we can use a spherical lens. In contrast to the cylindrical lens, a spherical lens supports the Fourier transformation of the incident beam in the radial direction. The intensity distribution in the focal plane is a Gaussian spot with radial symmetric properties. The transmittance function of a spherical lens can be written as

2 $$T(x)=\exp (i{k}_{0}(\frac{{x}^{2}}{2f}+\frac{{y}^{2}}{2f}))$$

where, $x$ and $y$ denotes the transverse coordinates, ${k}_{0}$ is the wave number and $f$ shows the focal length of the lens^2^.

### Bessel and Airy Beams

Other types of beams for SPIM are non-diffracting beams such as the Bessel beam and the Airy beam. The Bessel beam can be generated with conical superposition of the plane waves. The angular spectrum of a Bessel beam can be approximated by a ring, because the Fourier transformation of a ring with inner radius ${R}_{i}$ and outer radius ${R}_{0}$ is the zero order of a Bessel function. Thus, the Bessel beam can be created at the front focal plane of a Fourier lens, where an annular mask is placed in the back focal plane of it. The annular aperture can be defined with this amplitude mask:

3 $$A(r)=H[circ(\frac{r}{{R}_{0}})-circ(\frac{r}{{R}_{i}})].$$

The $H$ denotes the Heaviside function, $circ$ is the circular function, $r$ is radial transverse coordinate, ${R}_{0}$ and ${R}_{i}$ demonstrate the outer and inner radius of aperture, respectively^21^. The purity of Bessel beam can be determined by the size of the inner and outer radius of the annular mask, wavelength and the numerical aperture of the Fourier lens.

An Airy beam can be generated in one- or in two-dimensional shapes. Theoretically, the Fourier transformation of the optical beam with a Gaussian amplitude and a cubic phase produces an Airy beam. A one-dimensional (1D) Airy beam can be produced, when the cubic phase has just a k-vector in one direction (${k}_{x}^{3}$ or ${k}_{y}^{3}$). When two cubic phase maps are superimposed in two directions (${k}_{x}^{3}+{k}_{y}^{3}$), a two-dimensional (2D) Airy beam can be obtained. Therefore, the transmittance function of an element for producing an Airy beam can be written as,

4 $$T(x,y)=\exp (i\alpha {k}_{0}({k}_{x}^{3}+{k}_{y}^{3})),$$

where the dimensionless parameter $\alpha$ represents the cubic phase coefficient, which defines the property of the Airy beam; the invariant propagation length of the Airy can be controlled by changing the cubic phase coefficient $\alpha$^22^.

### Dammann Grating

An array generator is an optical element that splits the incoming beam into equal intensity beams. One of the most powerful methods to generate an array beam is using Dammann grating. Dammann grating is a binary phase grating that works like a beam splitter in one or two-dimensions. Assuming that all the optical elements have a transmission function, the transmission function of a Dammann grating has two values −1 and +1 corresponding to the phase values 0 and pi. Dammann grating is described with transitions points ${x}_{1}$, ${x}_{2}$, … ${x}_{n}$ related to the number of the desired intensity spots. A sufficient number of transitions per period of grating is critical in creating a specified number of beams. In general, for generating N beams, one period of the grating needs a ${\rm{K}}=2{\rm{int}}[\frac{{\rm{N}}}{2}]+2\,$and ${\rm{K}}=2\,{\rm{int}}[\frac{2{\rm{N}}-1}{4}]+2$ (K is the transition number), for odd and even number spots, respectively. For this approach, we used the transition points that are calculated according to C. Zhou. *et al.*^13^. (Suppl. Table 1). Dammann grating creates an incoherent beam array and it does not control the relationship between the beams. Therefore, for generating a beam array without superposition, the period of the grating should be large enough. It should be noted that the efficiency of the Dammann grating in odd numbers is higher than the even numbers, resulting in the odd numbers to cover a wider range of applications.

### Optimal Grating

The optimal grating is another method for generating an array of beams with a uniform distribution^15^. The phase function of optimal grating is described as:

5 $$T(x)=ta{n}^{-1}(\frac{\sum _{m=-N}^{N}{\gamma }_{m}\,\sin [(\frac{2\pi s}{\lambda })mx+{\alpha }_{m}]}{\sum _{m=-N}^{N}{\gamma }_{m}\,\cos [(\frac{2\pi s}{\lambda })mx+{\alpha }_{m}]}),$$

where m is the diffraction order, x shows the transverse coordinate, $\gamma$ and $\alpha$ are relative phase and intensity parameters associated with different diffraction orders; the angular separation between the diffraction orders is associated with $s$^15,16^. This method is limited to the odd number of the array intensity. It can support a 3, 5, 7, 9 and 11 fan of the intensity. The space between the beams can be controlled by changing the period of the grating. In SSPIM, the phase and intensity parameters that were proven in^15^ were used (Suppl. Table 2).

### Lattice beam

An optical lattice represents a type of coherent array beam, which can be generated in one, two, and three dimensions. The SPIM method requires a 1D-like lattice beam as an illumination beam^8^. The 1D optical lattice can be employed for two modes of the light sheet, the scanning or the structured illumination (SIM) mode. This beam can be formed using a Bravais pattern^8^. The Bravais pattern is a known 2D lattice defined by the discrete translation operation written as:

6 $$U={c}_{1}{{\bf{a}}}_{{\bf{1}}}+{c}_{2}{{\bf{a}}}_{{\bf{2}}},$$

where ${c}_{1}$ and ${c}_{2}$ can get any integer value and ${{\bf{a}}}_{{\bf{1}}}$ and ${{\bf{a}}}_{{\bf{2}}}$ are the primitive vectors. The discrete point with this translation operation can make a periodic pattern. The shape of the periodic pattern is related to the size and orientation of the primitive vectors. In general, a Bravias 2D lattice contains five periodic structures including oblique, rectangular, cantered rectangular, square and hexagonal structures. The first step in generating an optical lattice is to select the parameters of the lattice. These parameters are included in the size and the spatial separation of the spots of the lattice. In the second step, an arbitrary bounding function confines the lattice in one dimension. Then the spatial frequency and the behaviour of the bound lattice is adjusted with an annular mask in the Fourier domain. In the SPIM setup, if the SLM is conjugated to the front focal plane of the objective lens, the SLM pattern is a binarised bound lattice. The SLM pattern is the combination of the annular mask and Fourier transformation of the bound lattice. The square and hexagonal patterns are commonly used as lattice patterns. The lattice beams can be used in different modes, the scanning or the SIM mode. These two modes can be used for two different reasons, the light confinement and the axial resolution. The optimum light confinement can be achieved by controlling the distances between the cores of the coherent Bessel beams (spatial separation of the spots of the lattice). On the other hand, the tail of the Bessel beams in some regions produce a destructive interference. Therefore, the out-of-focus light will be reduced by confining the light towards the center of the beam.

### Tiling method

High-resolution imaging over a large FOV was significantly improved by applying a tiling method^10,11^. In this method, the illumination beam with a minimum waist (high axial resolution) and a small FOV can be swept in the direction of the propagating illumination light. For this reason, we need an optical element like a tunable lens to rapidly swipe the focused light at different positions through the sample. In other words, the tunable lens is able to change the curvature of the incident phase map. Hence, the initial illumination beam such as a Gaussian, a Bessel or a lattice beam should be merged with two or more lens phase maps. As mentioned above, the phase map of a lens is written as:

7 $$T(x)=\exp (i{k}_{0}(\frac{{x}^{2}}{2f}+\frac{{y}^{2}}{2f}))$$

In order to change the position or tiling of the focused light, the focal length of the lens needs to be changed. If we assume, we need three steps tiling to cover the specific FOV, we should select three different focal lengths (${f}_{1},{f}_{2},{f}_{3}$). Then, we will have three different phase maps (${T}_{1},{T}_{2},{T}_{3}$) which are merged with the initial phase map. In total, we will have three phase maps or phase patterns, which are loaded into the SLM. Each of these beams covers one-third of the field of view. Hence, in total we will have three images from one focus plane which then will be merged.

### SSPIM availability

SSPIM and the associated documentation are available at https://github.com/aakhtemostafa/SSPIM. The toolbox runs under the MATLAB computational environment or as standalone on Windows and MacOS.

## Electronic supplementary material

Method to engineer an SLM pattern for the 2D Airy beam

Method to engineer an SLM pattern for an incoherent Gaussian beam array.

Method to engineer an SLM pattern for an incoherent Bessel beam array.

Method to engineer an SLM pattern for a coherent square lattice beam.

Method to engineer an SLM pattern for coherent hexagonal lattice beams.

Method to engineer an SLM pattern for a coherent lattice beam.

Supplementary Materials 1
