# Supplementary material for: SSPIM: a beam shaping toolbox for structured selective plane illumination microscopy
Source: Sci Rep. 2018 Jul 3;8:10067. doi: 10.1038/s41598-018-28389-8 (PMC6030192; doi:10.1038/s41598-018-28389-8)
Supplement: Supplementary file 7 — Supplementary Materials 1 [file 41598_2018_28389_MOESM7_ESM.pdf]

Supplementary information for

SSPIM: a beam shaping toolbox for structured selective plane illumination microscopy

Mostafa Aakhte[1], Ehsan A. Akhlaghi[2,3] , H.-Arno J. Müller[1]

[1]Institute for Biology, Division of Developmental Genetics, University of Kassel, Heinrich-Plett Str. 40, 34132 Kassel, Germany

[2]Department of Physics, Institute for Advanced Studies in Basic Sciences (IASBS), Zanjan 45137-66731, Iran

[3]Optics Research Center, Institute for Advanced Studies in Basic Sciences (IASBS), Zanjan 45137-66731, Iran

**Supplementary Table S1**	**Parameters for binary value (0, π) Dammam grating for Array beam.**
**Supplementary Table S2**	**Parameters for gray value optimal grating for Array beam.**
**Supplementary Figure S1**	**Schematic of the openSPIM.**
**Supplementary Figure S2**	**Generation of the Gaussian beam with different circular masks.**
**Supplementary Figure S3**	**Generation of Bessel beams with different annular masks.**
**Supplementary Figure S4**	**Method to generate 2D Airy beams with different phase coefficient.**
**Supplementary Figure S5**	**Method to generate different number of incoherent array Gaussian beams.**
**Supplementary Figure S6**	**Method to generate different number of incoherent array Bessel beams.**
**Supplementary Figure S7**	**Method to generate different square patterns of coherent lattice beam.**
**Supplementary Figure S8**	**Method to generate different hexagonal patterns of coherent lattice beam.**
**Supplementary Figure S9**	**Method to generate and tile various type of beams.**
**Supplementary Movie 1**	**Method to engineer a SLM pattern for 2D Airy beam.**
**Supplementary Movie 2**	**Method to engineer a SLM pattern for incoherent array of Gaussian beams.**
**Supplementary Movie 3**	**Method to engineer a SLM pattern for incoherent array of Bessel beams.**
**Supplementary Movie 4**	**Method to engineer a SLM pattern for coherent square lattice beam.**
**Supplementary Movie 5**	**Method to engineer a SLM pattern for coherent hexagonal lattice beam.**
**Supplementary Movie 6**	**Method to engineer a SLM pattern for coherent lattice beam.**

Supplementary Table S1: Parameters for binary value (0, π) Dammam grating for Array beam. (first column) number of the array beam**. (second column)** Transition values for "n" copies of the beam.

Array Number	Transition Points
2	0.5
3	0.73526
4	0.22057, 0.44563
5	0.03863, 0.39084, 0.65552
6	0.11444, 0.20897
7	0.23191, 0.42520, 0.52571
8	0.06185, 0.17654, 0.20858, 0.31797
9	0.06668, 0.12871, 0.28589, 0.45666, 0.59090
10	0.10838, 0.11857, 0.19679, 0.23559, 0.31524, 0.37300
11	0.15015, 0.36389, 0.54103, 0.55344, 0.71318, 0.76612, 0.91107
12	0.01969, 0.08713, 0.12696, 0.18922, 0.24877, 0.35609
13	0.17765, 0.31352, 0.41244, 0.49846, 0.57633, 0.70857, 0.73041
14	0.05199, 0.12988, 0.19138, 0.24210, 0.27299, 0.31191, 0.35922, 0.49499
15	0.18240, 0.27424, 0.58581, 0.67967, 0.71917, 0.82217, 0.90642
16	0.14083, 0.17569, 0.22147, 0.26700, 0.35651, 0.39536, 0.43934, 0.45235
17	0.12271, 0.36812, 0.38508, 0.46844, 0.56010, 0.72061, 0.80310, 0.86000, 0.93712
18	0.03713, 0.06531, 0.11324, 0.11804, 0.13544, 0.18221, 0.25235, 0.39999, 0.42891, 0.46460
19	0.08570, 0.14057, 0.35809, 0.44535, 0.50140, 0.50781, 0.61168, 0.65143, 0.74179, 0.89057, 0.93947
20	0.04610, 0.15487, 0.17904, 0.21314, 0.24548, 0.27122, 0.30407, 0.38182, 0.39422, 0.44093
21	0.07505, 0.22656, 0.47886, 0.53661, 0.61683, 0.63009, 0.68194, 0.73870, 0.80083, 0.84469, 0.92196

Supplementary Table S2: Parameters for gray value optimal grating. (first row) Grating values for 3 beams. **(second row)** Grating values for 5 beams. **(third row)** Grating values for 7 beams. **(fourth row)** Grating values for 9 beams. **(fifth row)** Grating values for 11 beams.

Array Number	Diffraction Order	-5	-4	-3	-2	-1	0	1	2	3	4	5
3	μ_3					1.39	0	1.39				
	α_3					-pi/2	0	-pi/2				
5	μ_5				0.899	0.459	0	0.459	0.899			
	α_5				pi	-pi/2	0	-pi/2	pi			
7	μ_7			1.24	1.45	1.28	0	1.28	1.45	1.24		
	α_7			7.03	1.89	-0.99	0	-0.99	1.89	7.03		
9	μ_9		1.03	0.943	0.963	0.971	0	0.971	0.963	0.943	1.03	
	α_9		1.41	3.03	5.57	0.720	0	0.720	5.57	3.03	1.41	
11	μ_{11}	1.28	1.43	1.48	1.30	1.21	0	1.21	1.30	1.48	1.43	1.28
	α_{11}	4.41	5.55	2.85	4.49	0.311	0	0.311	4.49	2.85	5.55	4.41

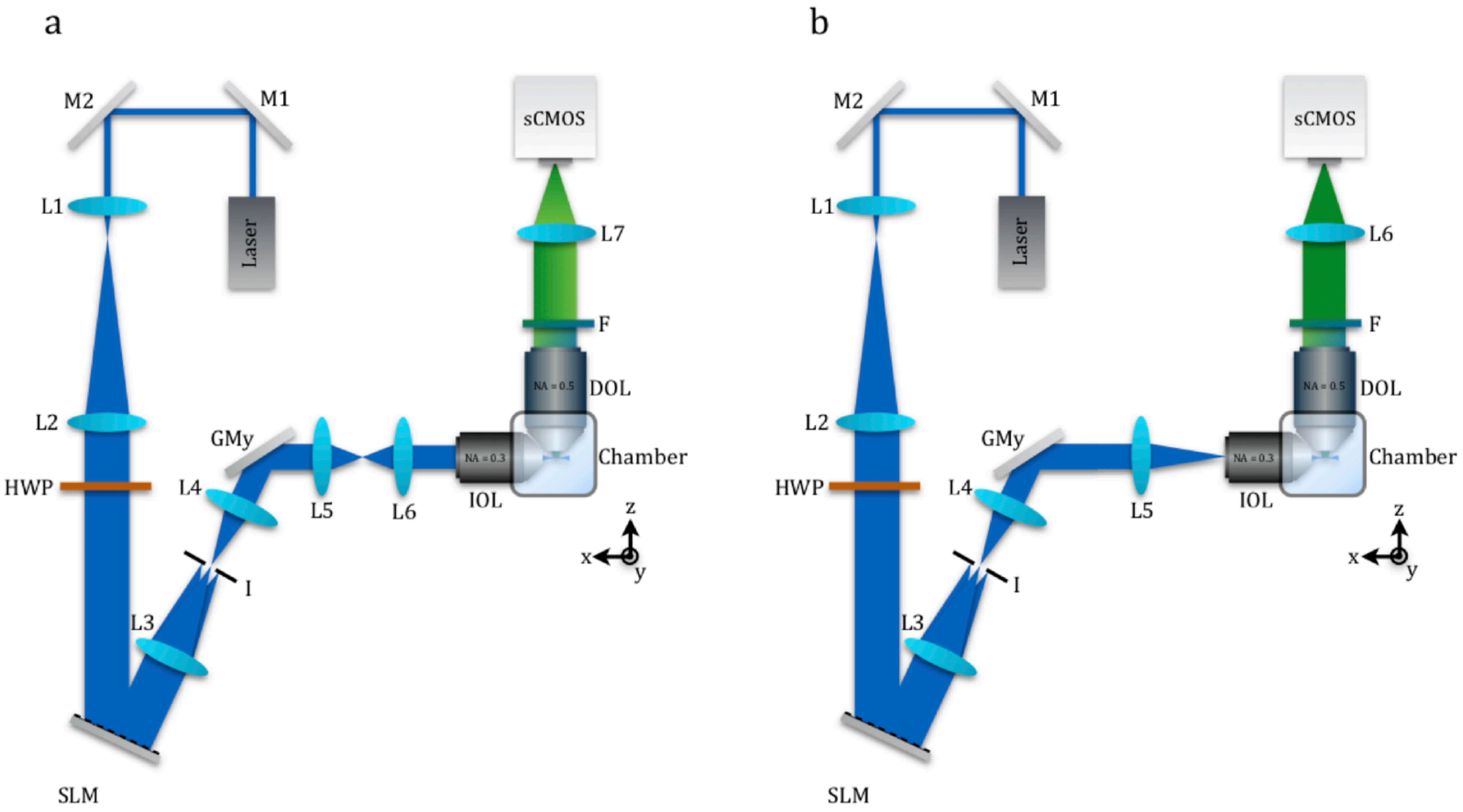

Supplementary Figure S1: Schematic of the OpenSPIM. (a) The direction of the laser beam is controlled by two mirrors (M1 and M2). The laser beam is then expanded and collimated using a pair of lenses (L1=25 mm, L2=300 mm). The laser beam has a linear polarization. Hence, the polarization orientation of the beam can be controlled with a half wave plate (HWP). The beam illuminates a ferroelectric spatial light modulator (SLM), upon which the desired pattern is projected. An Iris was placed at the focal plane of the lens L3 to block the undesired diffraction orders of the excitation light generated by the SLM (L3=150 mm, L4=75 mm). The conjugation of the SLM pattern is created with the lens L5 at a galvo mirror (GMy). It then passes through a pair of demagnification lenses (L6 = 50 mm and L7 = 25 mm) and is sent to an illumination objective lens (IOL). The Fourier transformation of the SLM pattern is achieved at the sample plane. The beam is oscillated in y direction with the GMy for creating a virtual light sheet. A narrow plane (x-y plane) of the sample is excited with the light sheet, which is imaged with a detection objective lens (DOL), fluorescence emission filter (F), a tube lens (L8) and the sCMOS in orthogonal direction. **(b)** The overall view of this setup represents the setup in (**a**), although the SLM is conjugated into the front focal plane of the IOL meaning that the SLM pattern is imaged into the front focal plane of the IOL.

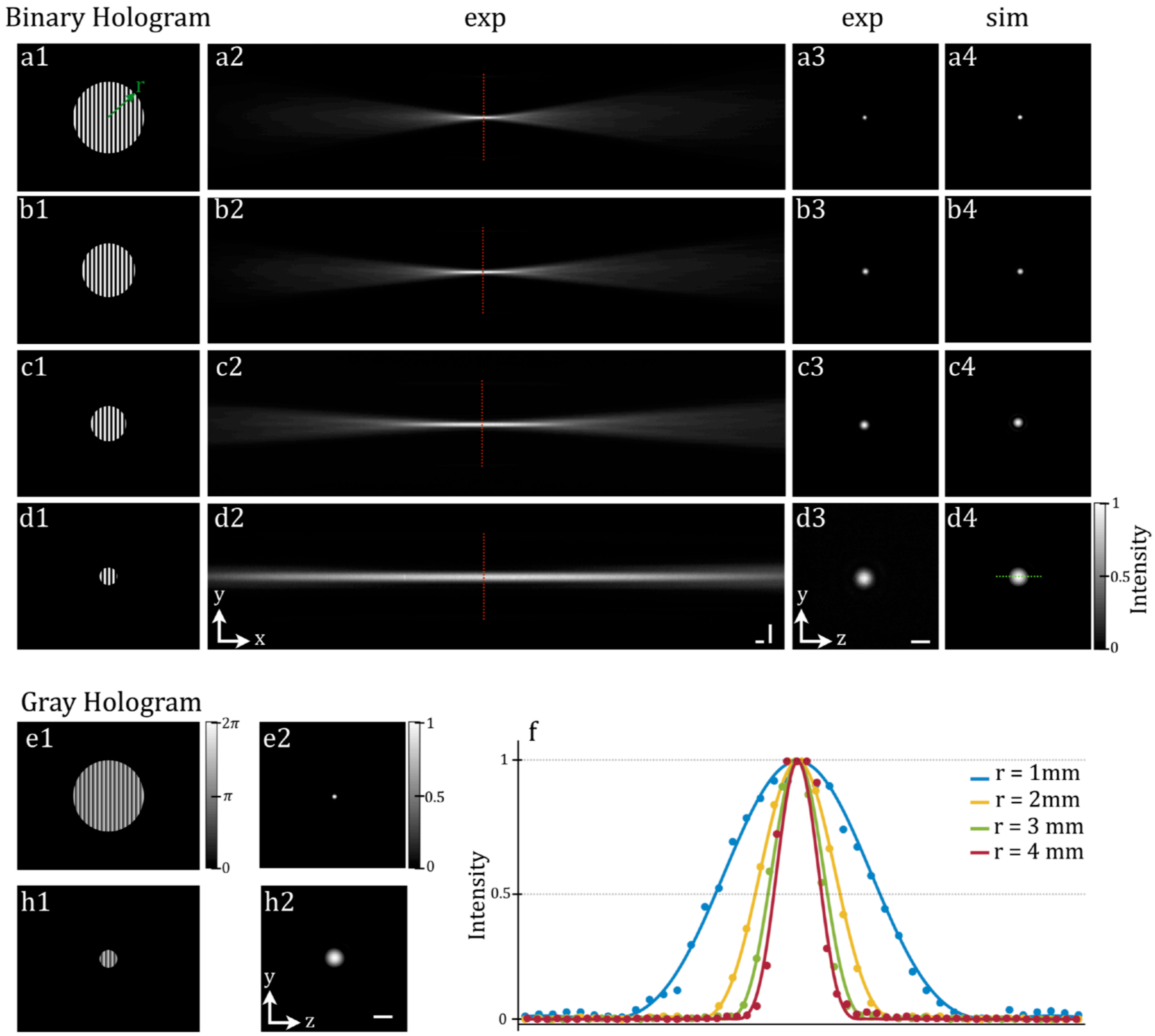

Supplementary Figure S2: Generation of a Gaussian beam with different circular masks. (a1-d1) The binary SLM pattern for generating different Gaussian beams are depicted. The radii (r) of the circular masks are changed from 4 mm to 1 mm. Vertical stripes represent the binary grating for separating the Gaussian beam of inherent SLM diffraction pattern. **(a2-d2)** Measurements of the propagation intensity through the dye solution. **(a3-d3)** Measurements of the cross-section intensity of the beams. **(a4-d4)** Cross-section intensities of the simulated beams for binary holograms. **(e1, h1)** Gray value holograms for generating different Gaussian beams. **(e2-h2)** Quantitative comparison of the cross sections of Gaussian beams with different numerical apertures that are shown in **(a3-d3** and **a4-d4).** The solid lines and the circular spots show the simulation (sim) and experimental (exp) data, respectively. Scale bars, 20µm.

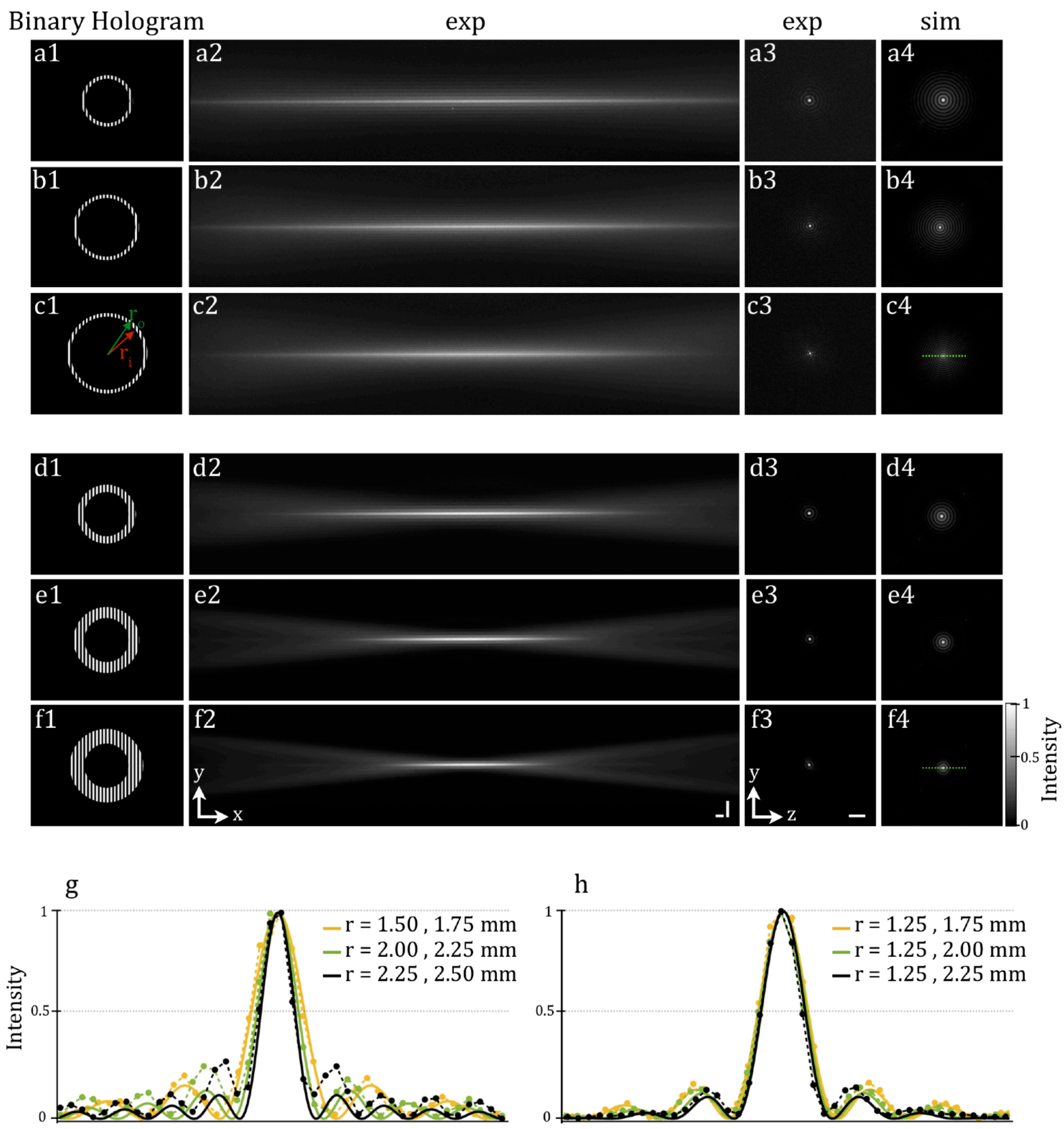

Supplementary Figure S3: Generation of Bessel beams with different annular masks. (a1, b1 and **c1)** The binary SLM pattern for generating different Bessel beams are depicted. The inner radii (r_i) of the annular masks are changed from 1.5 to 2.5 mm; the outer radii (r_o) are changed simultaneously from 1.75 to 2.75 with 0.25 mm step size. **(d1,e1** and **f1)** depict binary SML pattern for generating different Bessel beams. The inner radii of the annular masks remain constant (r_i = 1.25 mm), but the outer radii (r_o) are changed from 1.75 to 2.25 with 0.25 mm step size. **(a2-f2,** second column**)** The propagation intensity through the dye solution was measured. **(a3-d3,** third column**)** Measurement of the cross-section intensity of the beams. **(a4-d4** fourth column**)** Cross-section intensities of the simulated beams for binary holograms. **(g,h)** Quantitative comparison of cross section of Bessel beams with different annular masks are shown in **(a3-c3** and **a4-c4)** and **(d3-f3** and **d4-f4).** The solid lines and circular spots with the dashed lines show the simulation and experimental data, respectively. Scale bars, 20µm.

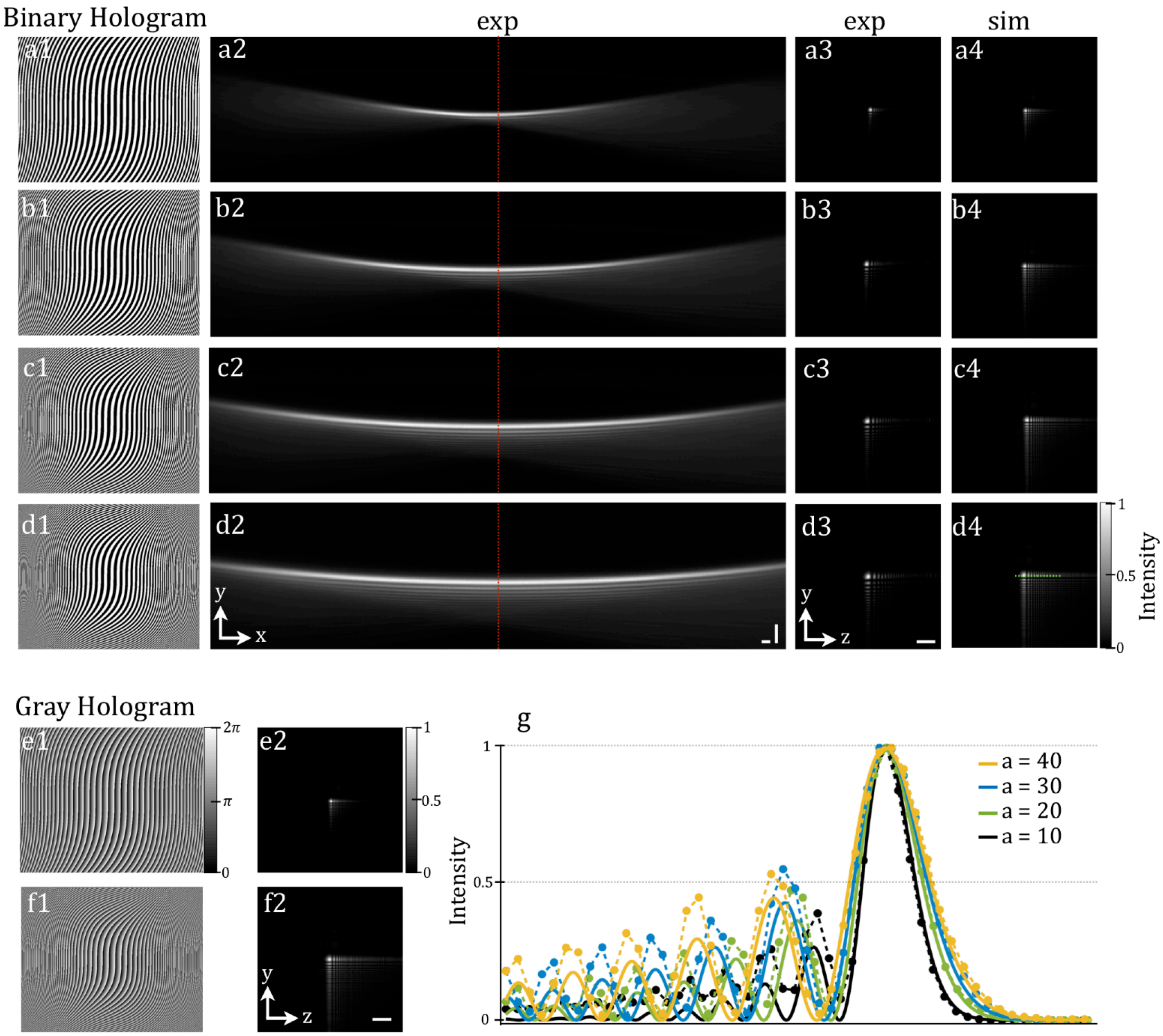

Supplementary Figure S4: Method to generate 2D Airy beams with different phase coefficients. (a) (a1-d1) Binary SML pattern for generating Airy beams with different cubic phases. The cubic phase coefficient is changed from 10 to 40, respectively. **(a2-d2)** Measurement of the propagation of Airy beams through the dye solution. **(a3-d3)** Measurement of the cross section intensity of the beams. **(a4-d4)** Cross-section intensities of the simulated beam arrays. **(e1-f1)** Gray value SLM pattern for generating Airy beams. **(e2-f2)** Cross-section intensities of the simulated beam arrays. **(g)** Quantitative comparison of cross sections of Airy beams with different cubic phases that are shown in **(a3-d3** and **a4-d4).** The solid lines and the circular spots with the dashed lines show the simulation (sim) and experimental (exp) data, respectively. Scale bars, 20µm.

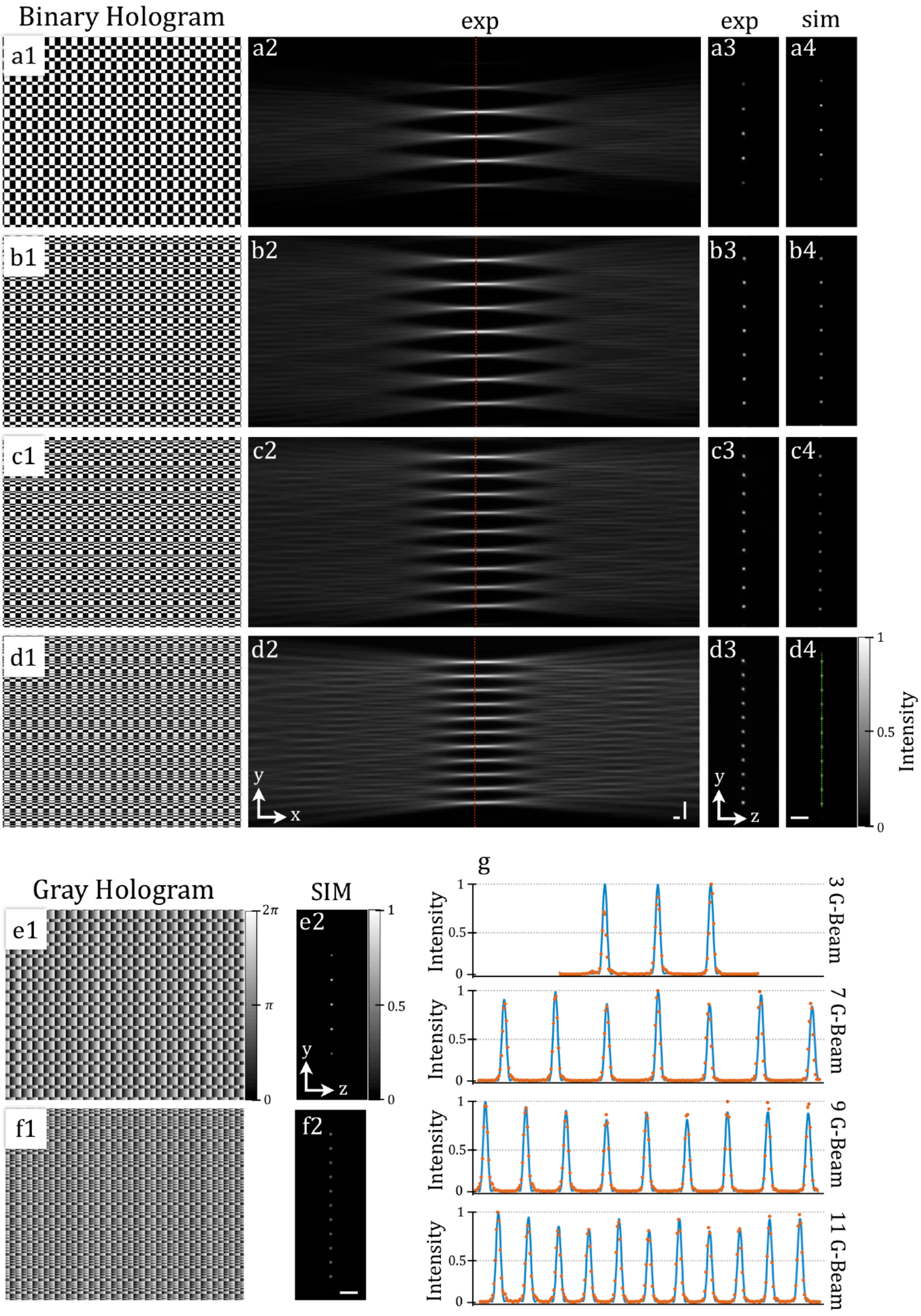

Supplementary Figure S5: Method to generate incoherent array Gaussian beams with different numbers of individual beams. (a1-d1) Binary SML patterns for generating 3, 7, 9 and 11 gaussian beam arrays (G-Beam). All of the SLM patterns are created with Dammann grating. The vertical and horizontal stripes demonstrate the binary grating and the Dammann grating, respectively. **(a2-d2)** Propagation of the beam arrays obtained in a dye solution. **(a3-d3)** Measurement of the cross-sections of beam arrays. **(a3-d3)** Cross-sections of the simulated beam arrays. **(e1-f1)** Gray value blazed phase added to the binary Dammann grating for generating 3, 7, 9 and 11 Gaussian beam arrays, respectively. **(e2-h2)** Cross-sections of the simulated beam arrays using gray value phases. Quantitative comparison of the cross sections of Gaussian beam array are shown in **(a3-d3** and **a4-d4)**. The solid lines and the circular spots show the simulation (sim) and experimental (exp) data, respectively. Scale bars, 20μm.

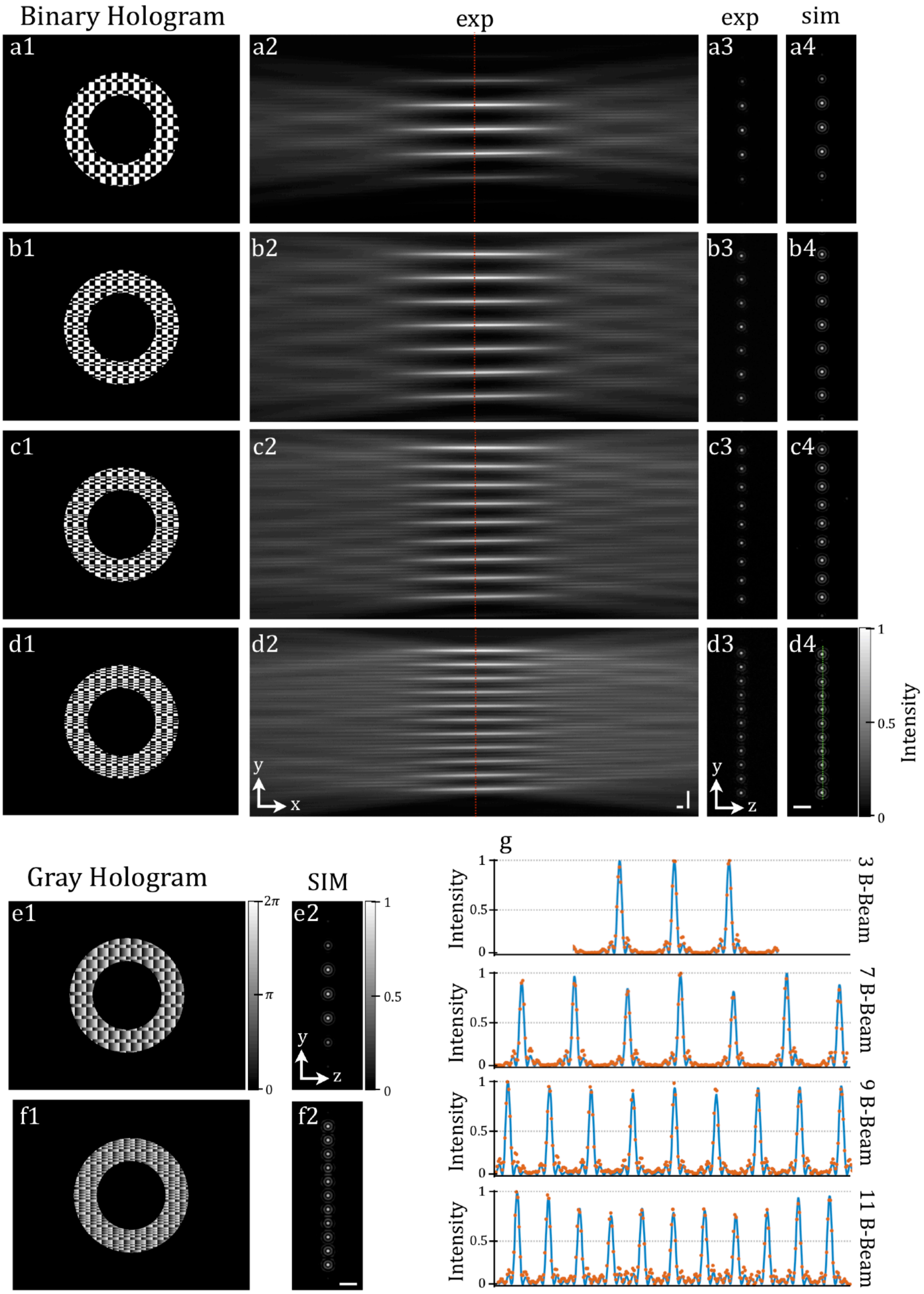

Supplementary Figure S6: Method to generate incoherent array Bessel beam arrays composed of different number of individual beams. (a1-d1) Binary SLM pattern for generating 3, 7, 9 and 11 array Bessel beams (B-Beam). All of the SLM patterns are created with Dammann grating. The vertical and horizontal stripes demonstrate the binary grating and the Dammann grating, respectively. **(a2-d2)** Propagation of the beam arrays obtained in a dye solution. **(a3-d3)** Measurement of the cross-section of beam arrays. **(a3-d3)** Cross-sections of simulated beam arrays. **(e1-f1)** Gray value blazed phase added to binary Dammann grating for generating 3, 7, 9 and 11 Gaussian beam arrays. **(e2-f2)** Cross-sections of simulated beam arrays using gray value phases. Quantitative comparison of the cross sections of Bessel beam arrays are shown in **(a3-d3** and **a4-d4)**. The solid lines and circular spots show the simulation (sim) and experimental (exp) data, respectively. Scale bars, 20μm.

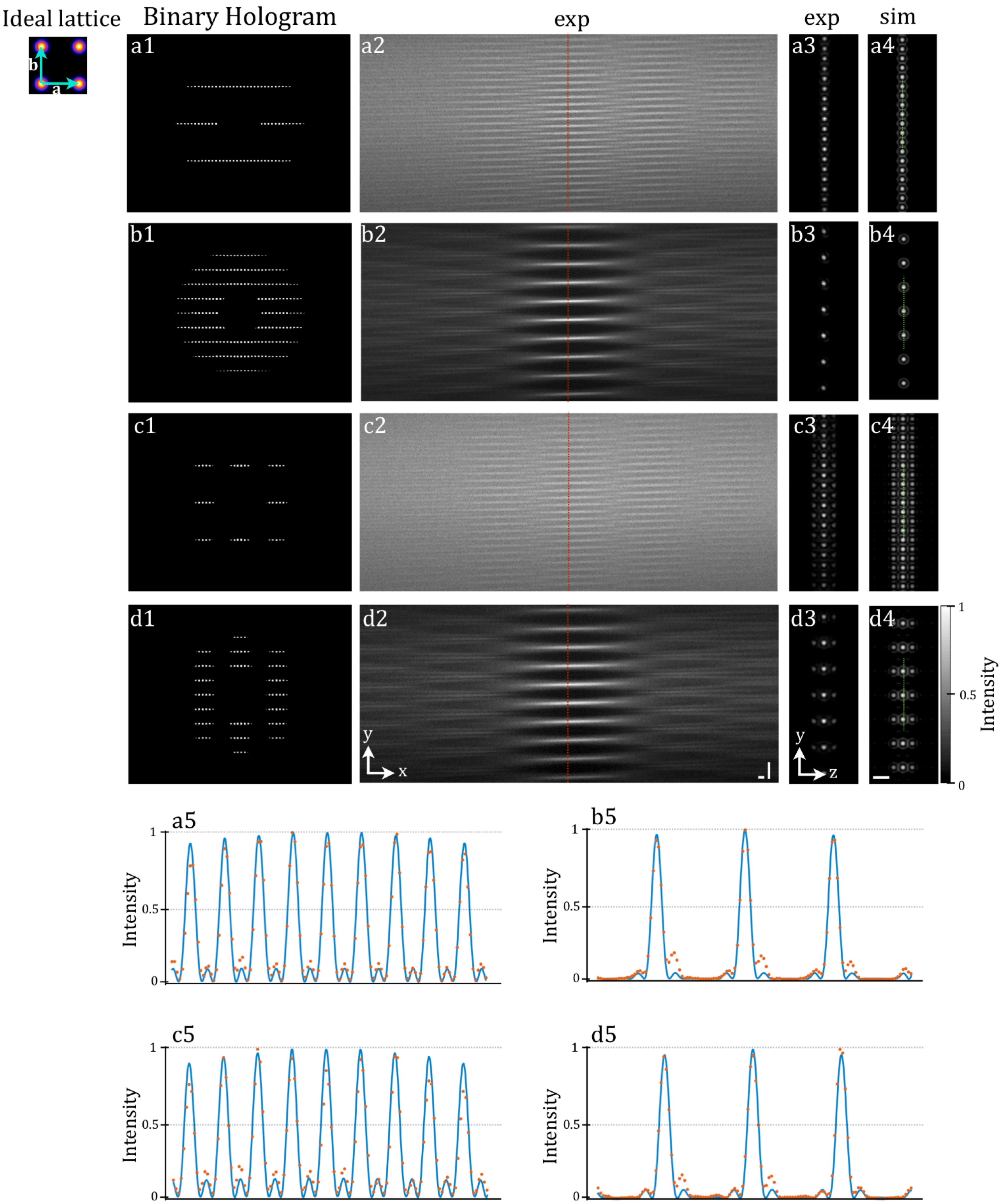

Supplementary Figure S7: Method to generate different square patterns of coherent lattice beams. (a1-b1) Binary SLM pattern for generating 1D square lattice with a narrow (0.1 mm) bounding function, and with variable separation of the spots in vertical direction (b=0.2 and 0.5 mm, a=0.5 mm). **(c1-d1)** Binary SLM pattern for generating 1D square lattice with a thick (0.3 mm) bounding function, and different separation spots in vertical direction (0.2 and 0.5 mm). **(a2-d2)** Propagation of lattice beams as obtained in a dye solution. **(a3-d3)** Measurements of the cross-sections of the lattice beams. **(a4-d4)** Cross-sections of the simulated beam arrays. **(a5-d5)** Quantitative comparison of the cross sections of the lattice beams are shown in **(a3-d3** and **a4-d4)**. The solid lines and circular spots show the simulation (sim) and experimental (exp) data, respectively. Scale bars, 20µm.

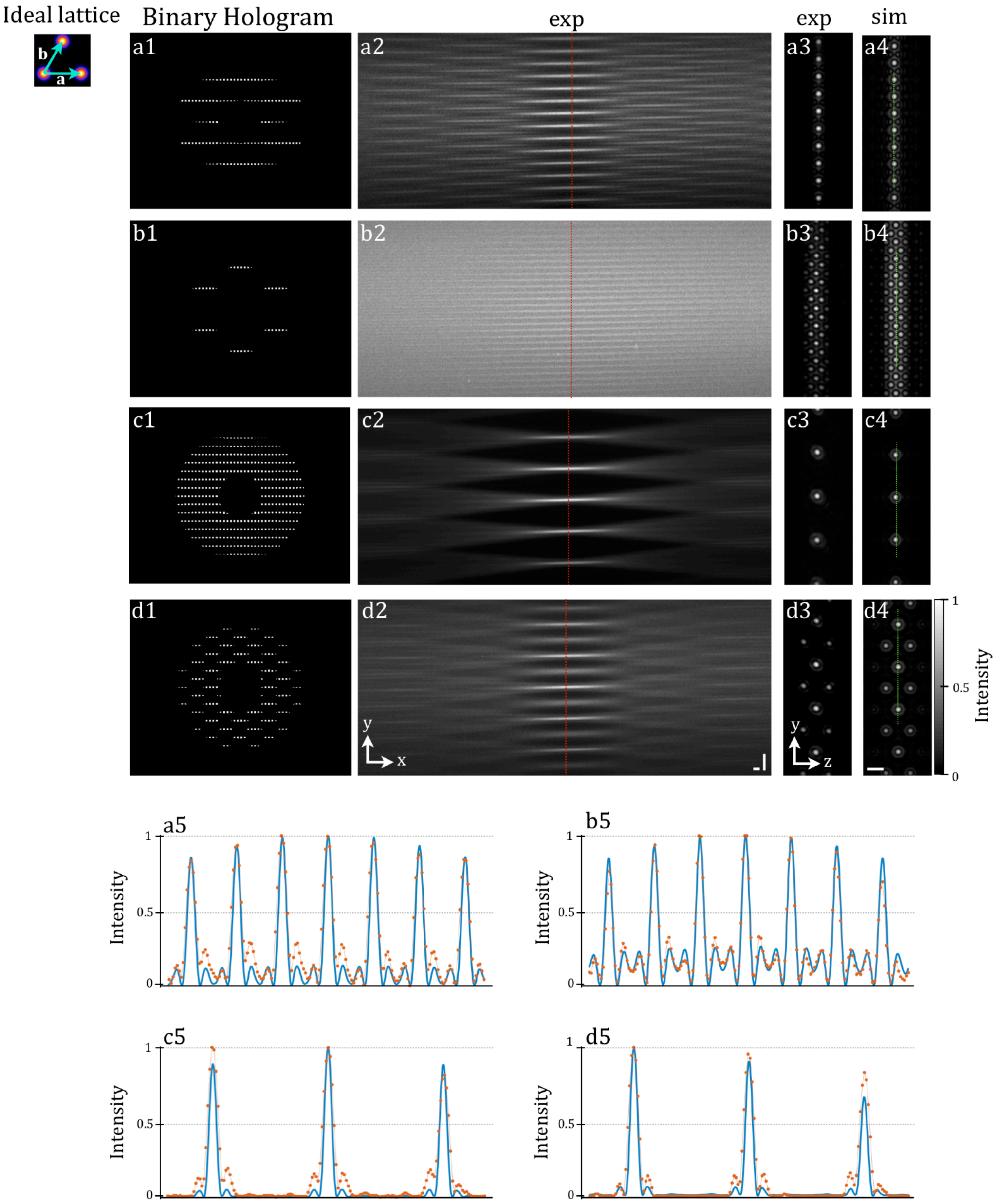

Supplementary Figure S8: Method to generate different hexagonal patterns of coherent lattice beams. (a1-b1) Binary SLM patterns for generating a 1D hexagonal lattice beam with a narrow (0.1 mm) and thick (0.3 mm) bounding function (separation spots: b = 0.2 and a = 0.2 mm). **(c1-d1)** Binary SLM patterns for generating 1D hexagonal lattice beams with a narrow (0.1 mm) and thick (0.3 mm) bounding function (separation spots: b = 0.5 and a = 0.5 mm). **(a2-d2)** Propagation of the lattice beam obtained in dye solution. **(a3-d3)** Measurement of cross-sections of experimental lattice beams. **(a4-d4)** Cross-sections of simulated lattice beams. **(a5-d5)** Quantitative comparison of cross sections of the lattice beams are show in **(a3-d3** and **a4-d4)**. The solid lines and the circular spots show the simulation (sim) and experimental data (exp), respectively. Scale bars, 20µm.

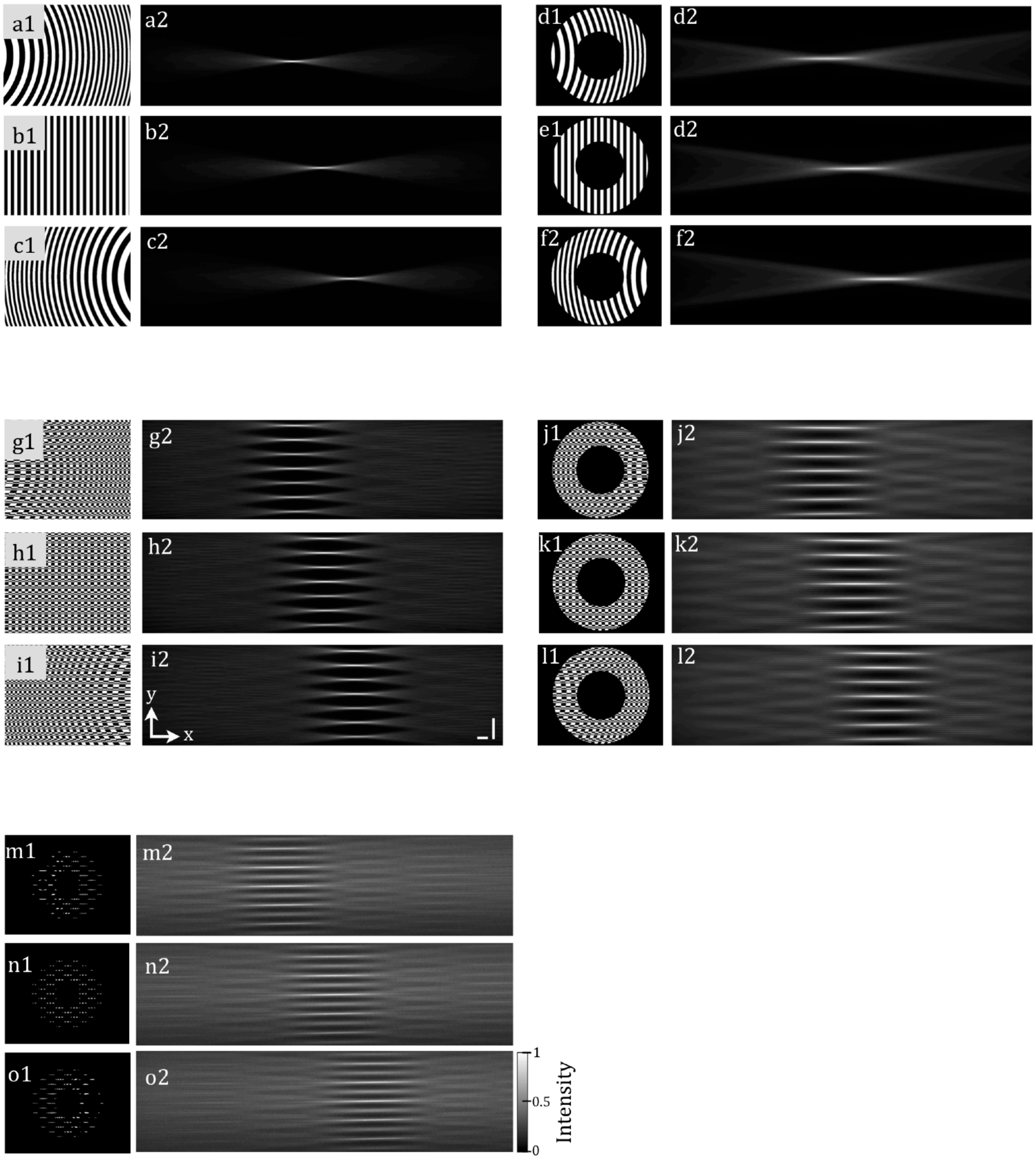

Supplementary Figure S9: Methods to generate tiled modes of the various types of beams. (a1-c1) Binary SLM pattern of the tiled single Gaussian beams. **(a2-c2)** Recorded profile of the tiled single Gaussian beam in a dye solution. **(b1-f1)** Binary SLM patterns of the tiled single Bessel beam (r_o = 3 mm, r_i = 2 mm). **(b2-f2)** Recorded profile of the tiled single Bessel beam in a dye solution. **(g1-i1)** Binary SLM pattern of the tiled 7 incoherent Gaussian beam array. **(g2-i2)** Recorded profile of the tiled 7 incoherent Gaussian beam array in a dye solution. **(j1-l1)** Binary SLM patterns of the tiled 7 incoherent Bessel arrays (r_o = 2.5 mm, r_i = 2 mm) beam. **(j2-l2)** Recorded profile of a tiled 7 incoherent Bessel beam array in the dye solution. **(m1-o1)** Binary SLM patterns of the tiled coherent lattice beams. **(m2-o2)** Recorded profile of the tiled lattice beams in a dye solution. Scale bars, 40μm.

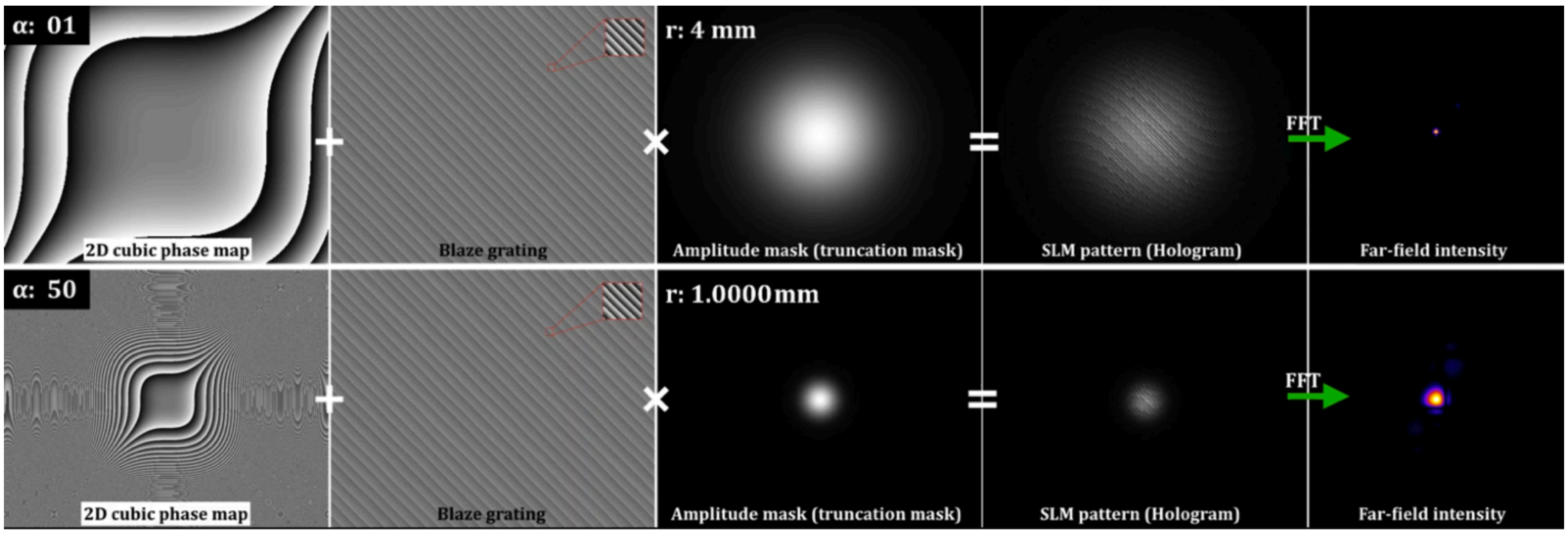

Supplementary Movie 1: Method to engineer an SLM pattern for the 2D Airy beam. (upper row) Increasing the phase coefficient (α) and its effects on the main lobe and invariant propagation length of the 2D Airy beam. The phase coefficient is changed from 1 to 50. **(lower row)** The number of the side lobes is controlled by changing the radius of the amplitude mask (truncation mask) for a constant α.

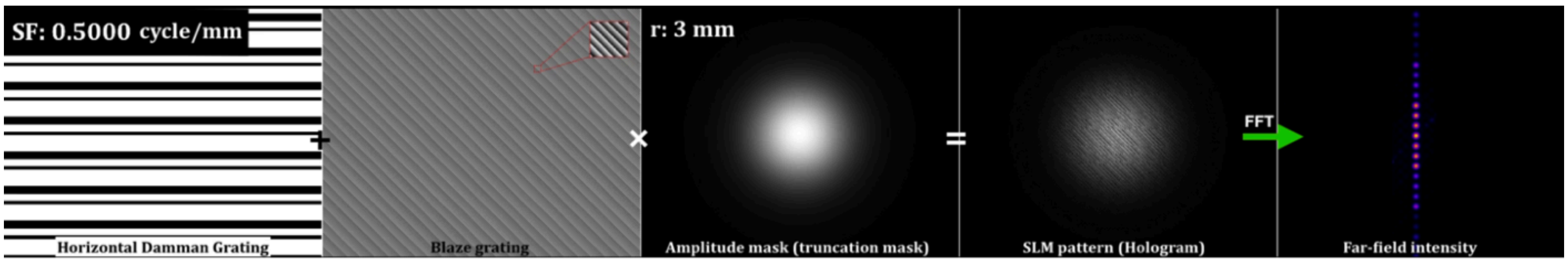

Supplementary Movie 2: Method to engineer an SLM pattern for an incoherent Gaussian beam array. In order to avoid the interference patten between the spots of an array beam, the spatial frequency of the Dammann grating should be adjusted. The spatial frequency (SF) of the binary Dammann grating is increased, hence, the separation angle between the spots will be increased.

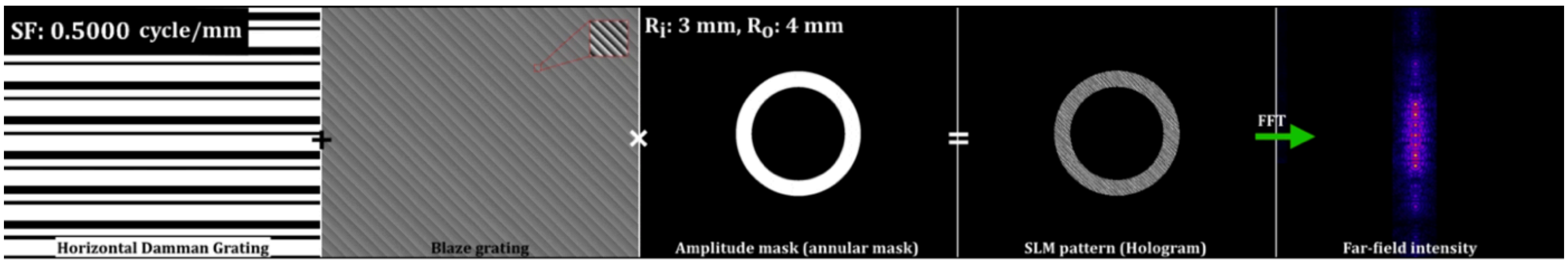

Supplementary Movie 3: Method to engineer an SLM pattern for an incoherent Bessel beam array. In order to avoid the interference pattern between the spots of an array beam, the spatial frequency of the Dammann grating should be adjusted. The spatial frequency (SF) of the binary Dammann grating is increased, hence, the separation angle between the spots will be increased.

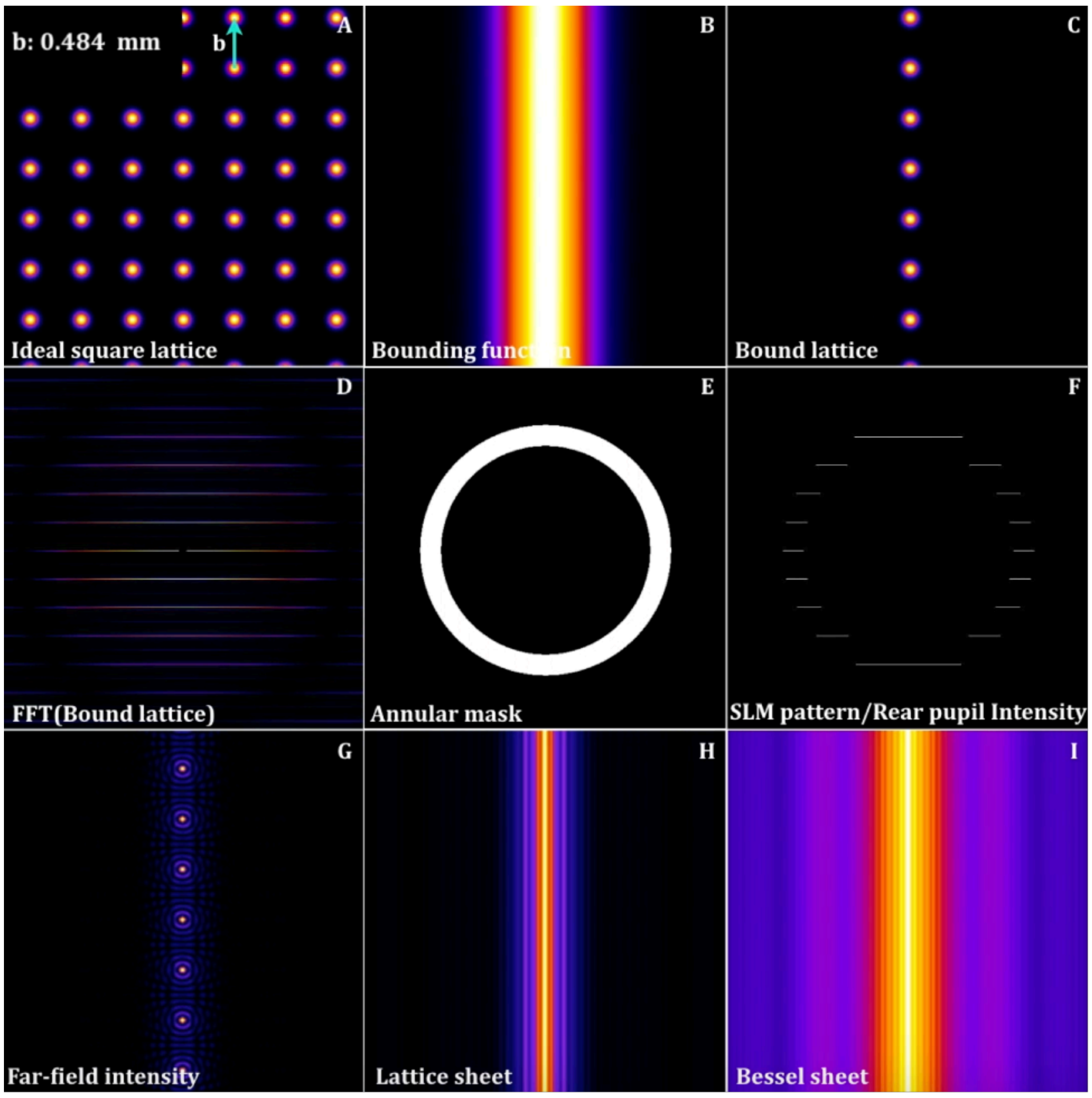

Supplementary Movie 4: Method to engineer an SLM pattern for a coherent square lattice beam. In order to find the maximum light confinement, the distance between the spots in the ideal lattice in the direction of the scanning (b) can be adjusted.

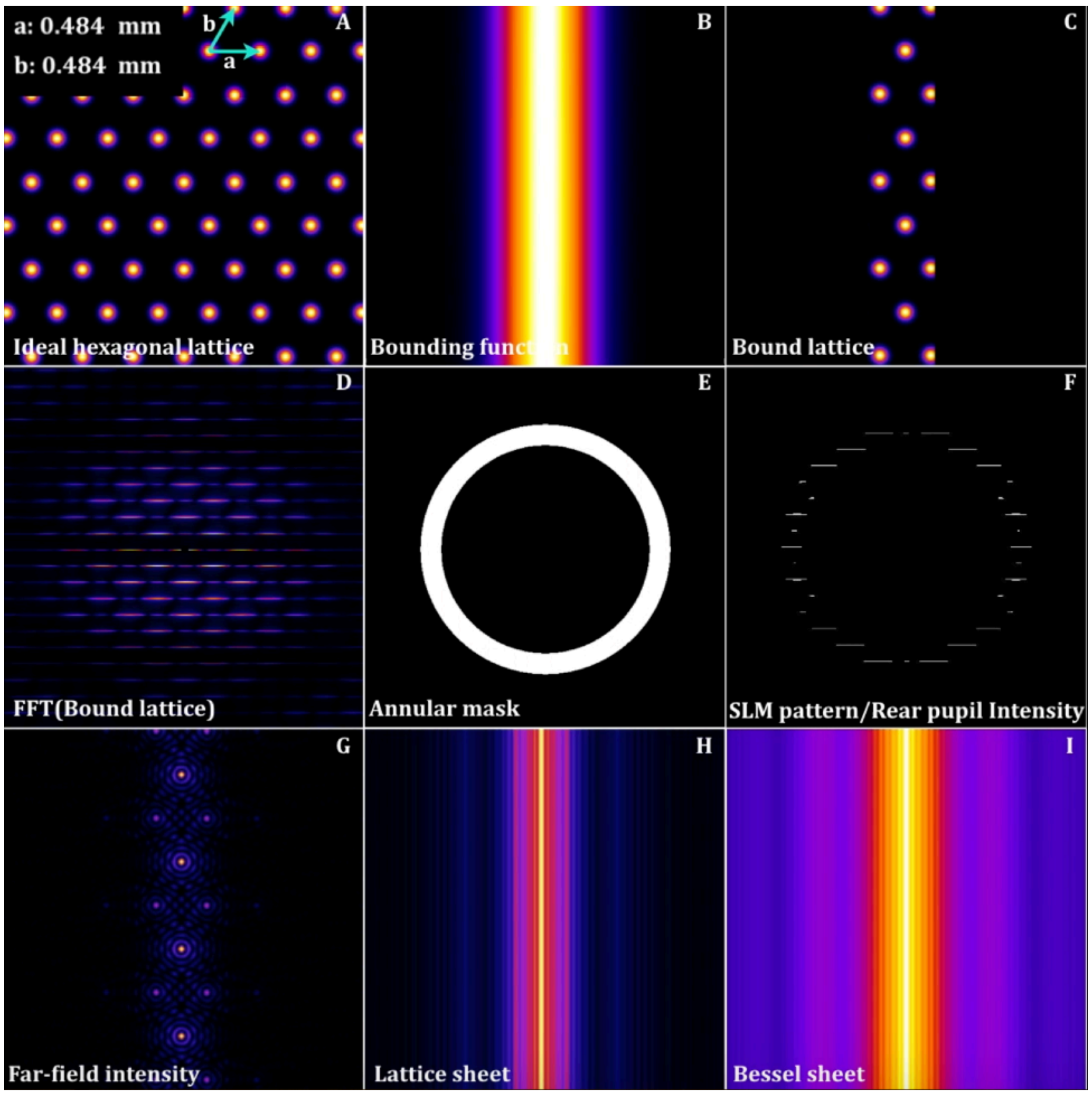

Supplementary Movie 5: Method to engineer an SLM pattern for coherent hexagonal lattice beams. In order to maximize the axial resolution, the distance between the spots in the ideal lattice (b and a) can be adjusted.

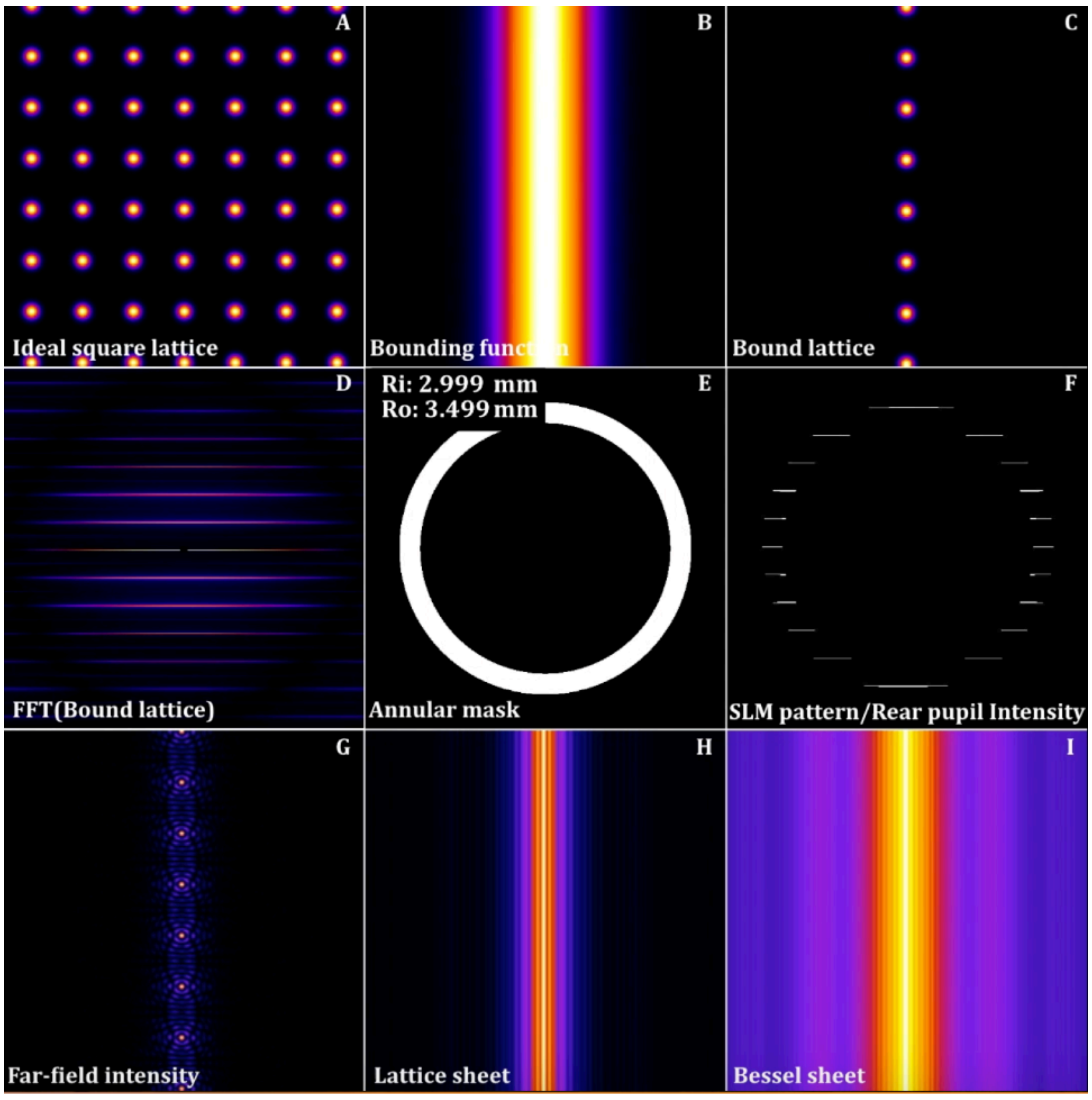

Supplementary Movie 6: Method to engineer an SLM pattern for a coherent lattice beam. The spatial frequency of the lattice beam can filtered with an annular mask. In the example presented in this movie, the inner and outer radius of the annular mask are decreased. The core size of the Bessel beams and the distance between them can be controlled.
